# The Role of CD28 and CD8^+^ T Cells in Keloid Development

**DOI:** 10.3390/ijms23168862

**Published:** 2022-08-09

**Authors:** Mengjie Shan, Hao Liu, Yan Hao, Kexin Song, Cheng Feng, Youbin Wang

**Affiliations:** 1 Peking Union Medical College, Chinese Academy of Medical Sciences, Peking Union Medical College Hospital, Beijing 100730, China; 2 Department of Plastic Surgery, Peking Union Medical College Hospital, Beijing 100730, China

**Keywords:** CD28, CD8^+^ T cells, immune microenvironment, keloid, neural network model

## Abstract

**Background**: A keloid is a benign skin tumor that extends beyond the initial injury area, and its pathologic mechanism remains unclear. **Method**: High-throughput sequencing data were obtained from normal skin tissue of patients with keloids (Group N) and healthy controls (Group C). Important genes were mined by bioinformatics analysis and identified by RT–qPCR, Western blotting, immunohistochemistry and immunofluorescence assays. The CIBERSORT algorithm was used to convert gene expression information into immune cell information. Flow cytometry was used to verify the key immune cells. Fluorescence-activated cell sorting coculture and CCK8 experiments were used to explore the effect of CD8^+^ T cells on keloid-associated fibroblasts. Neural network models were used to construct associations among CD28, CD8^+^ T cells and the severity of keloids and to identify high-risk values. **Result**: The expression levels of costimulatory molecules (CD28, CD80, CD86 and CD40L) in the skin tissue of patients with keloids were higher than the levels in healthy people (*p* < 0.05). The number of CD8^+^ T cells was significantly higher in Group N than in Group C (*p* < 0.05). The fluorescence intensities of CD28 and CD8^+^ T cells in Group N were significantly higher than those in Group C (*p* = 0.0051). The number and viability of fibroblasts cocultured with CD8^+^ T cells were significantly reduced compared with those of the control (*p* < 0.05). The expression of CD28 and CD8^+^ T cells as the input layer may be predictors of the severity of keloids with mVSS as the output layer. The high-risk early warning indicator for CD28 is 10–34, and the high-risk predictive indicator for CD8^+^ T cells is 13–28. **Conclusions**: The abnormal expression of costimulatory molecules may lead to the abnormal activation of CD8^+^ T cells. CD8^+^ T cells may drive keloid-associated immunosuppression. The expression of CD28 and CD8^+^ T cells as an input layer may be a predictor of keloid severity. CD28 and CD8^+^ T cells play an important role in the development of keloids.

## 1. Introduction

A keloid is an benign skin tumor that extends beyond the initial injury area and invades adjacent normal skin [1,2,3,4,5,6,7,8]. The most obvious pathological characteristic of keloids is the excessive and disordered deposition of collagen fibers [2,9,10,11,12]. Immune and inflammatory disorders in the wound healing process have been proposed by many authors [13,14,15,16,17,18]. Keloid fibroblasts are often considered an important cause of keloid formation, but regulation of the immune microenvironment of keloid fibroblasts has not been deeply explored. In the keloid immune microenvironment, an increase in M2 macrophage numbers and a decrease in M1 macrophage numbers appear to play an important role in keloid susceptibility [19]. Murao et al. [20] found that the presence of a large number of Tregs decreased the expression of type I collagen and TGF-β mRNA in keloids. Jin Q et al. [17] found that macrophages can promote Treg differentiation by upregulating Foxp3 expression. Macrophages in scar tissue are highly activated and polarized to the M2 subtype, and these macrophages promote Treg differentiation by upregulating Foxp3 expression. However, the role of CD8^+^ T cells in the keloid immune microenvironment is poorly studied.

The cytotoxicity of CD8^+^ T cells is the key to suppressing tumors [21,22]. Olivo Pimentel V et al. [23] found that CD8^+^ T cells killed approximately 50% of target tumor cells after 48 h of coculture with tumor cells. Kato T et al. [24] also found that CD8^+^ T cells and cancer-associated fibroblasts were negatively correlated in tumor tissue. Aghajanian et al. [25] found that cardiac fibroblasts that express a xenogeneic antigen can be effectively targeted and ablated by the adoptive transfer of antigen-specific CD8^+^ T cells.

Keloid-related fibroblasts have a strong proliferation ability, which is different from healthy fibroblasts and is also the reason for keloid proliferation. In this study, we sought to explore the role of CD28 and CD8^+^ T cells in keloid development based on an analysis of immune-related gene databases in patients with keloids.

## 2. Result

### 2.1. DEGs between Groups N and C

**Figure 1:**
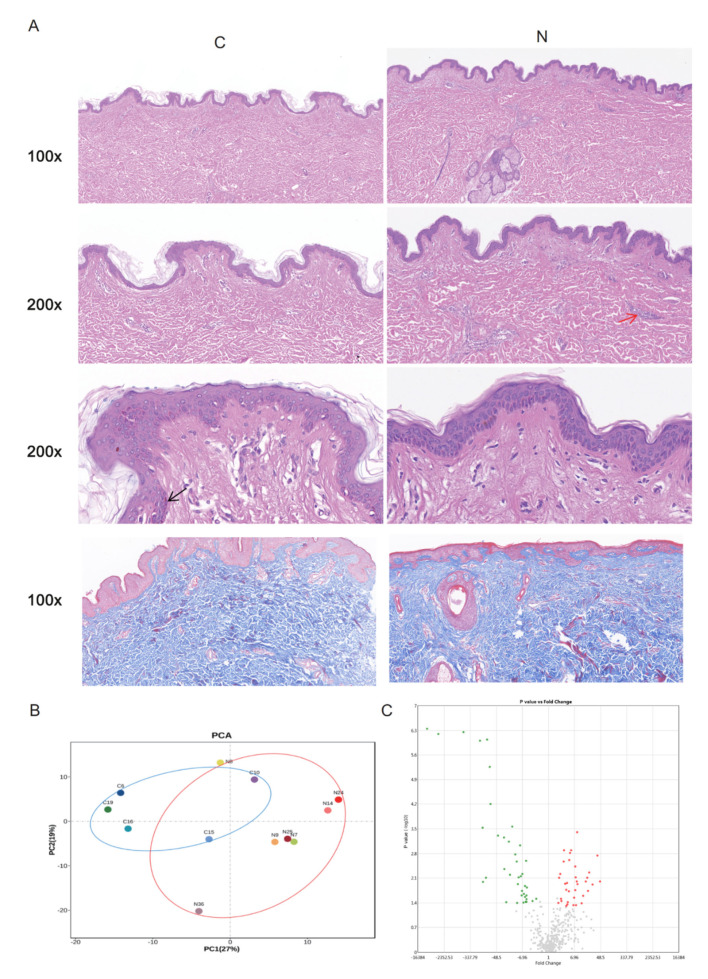
(**A**) Histological images of Group C and Group N, 100×, 200×, 400×, black arrow, pigment deposition; red arrow, microvessel. Masson stain, 100×. (**B**) Unsupervised principal component analysis (PCA) of Group C and Group N. Each point in the figure represents a sample, and the position of the point in the figure is determined by all the metabolites in the sample. The ellipse in the figure is based on the 95% confidence interval calculated and drawn by Hotelling T2. The sample falling outside the ellipse implies that the point may be an outlier. (**C**) The volcano plot illustrates the differences between Group C and Group N. Red represents up-regulated genes, and green represents down-regulated genes.

HE staining and Masson staining were used to identify the characteristics of normal skin tissue from cosmetic surgery patients (Group C) and normal skin tissue from patients with keloids (Group N) (Figure 1A). In HE-stained tissue, pigment deposition could be observed in the basal cells in the epidermis. Many microvessels could be observed in the loose connective tissue of the derma papillary layer. The dermal reticular layer mainly consisted of thick collagen fibers. There were no obvious HE-staining differences between the groups (Figure 1A).

All the included samples were divided into two groups on PC1 and PC2 dimensions (Figure 1B, Table 1). Although there was not much difference on a histology level, the samples of Groups N and C were obviously divided into clusters through dimension reduction analysis. Twenty-five genes were upregulated in Group C compared to Group N and 39 genes were downregulated in Group C compared to Group N (Figure 1C, Table S1).

### 2.2. Identification of Hub Genes

**Figure 2:**
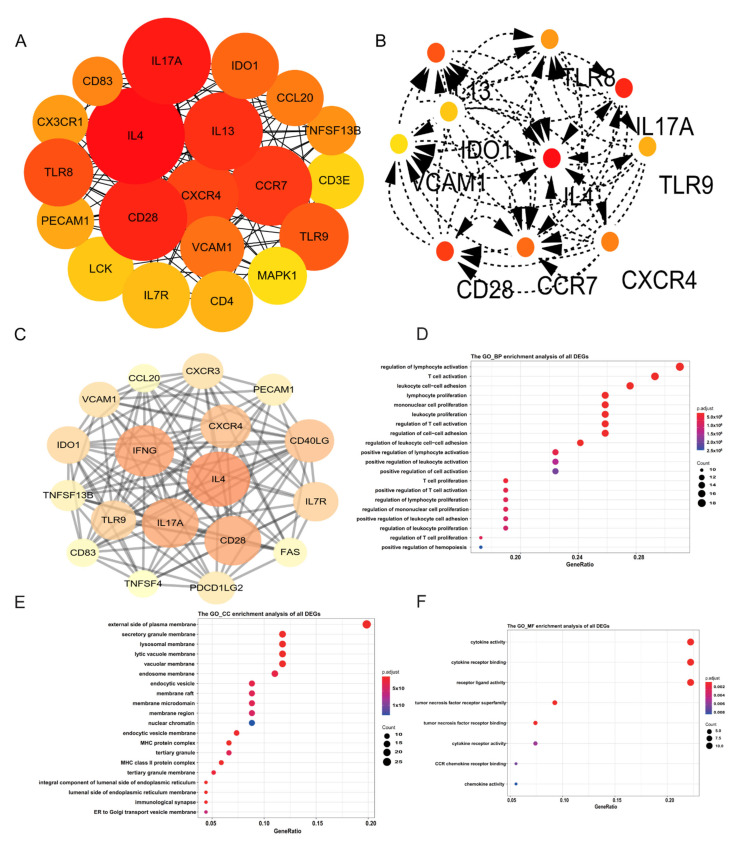
(**A**) Protein–protein interaction (PPI) network of differentially expressed genes (DEGs). (**B**) The cytoHubba algorithm was also used to screen hub genes from the PPI network. (**C**) The MCODE algorithm was used to screen the differentially expressed genes and obtain the important genes involved in pathogenesis. (**D**–**F**) Detailed information relating to changes in the biological processes (BP), cellular components (CC) and molecular functions (MF) of DEGs in Group C and Group N through GO enrichment analyses.

To explore the immune response in the early stage of keloids, patients with keloid onset less than 5 years ago were included in the study. Based on differentially expressed genes, the contribution degree of a node in the whole network can be determined by the number of connections between the node and other nodes. DEGs were imported into Cytoscape to construct PPIs to clarify the interactions between molecules (Figure 2A). The type and intensity of interactions between coding genes are presented in PPI. Genes such as IL-4, IL-13, CD28 and CCR7 were highlighted in the network diagram. A degree > 10 was set as further filtering. IL-13, TLR8, VCAM1, IDO1, IL-4, IL-17A, CD28, CCR7, CXCR4 and TLR9 were the 10 hub genes ultimately screened from the PPI network (Figure 2B); These genes may serve as the target genes for the pathogenesis of keloids, which need further verification. The MCODE plug-in of Cytoscape was used to filter important modules in the PPI network (Figure 2C).

### 2.3. Functional Annotation of DEGs

**Figure 3:**
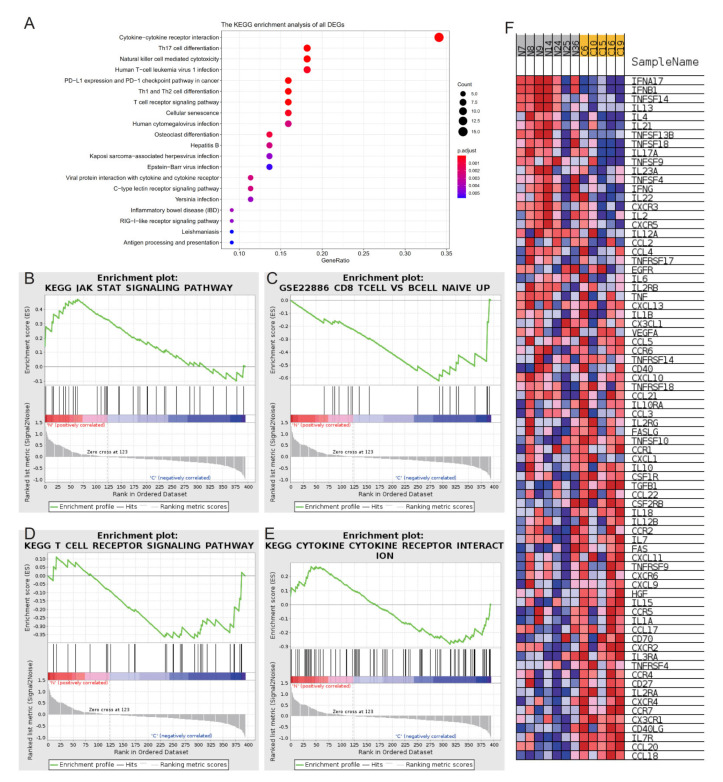
(**A**) KEGG analysis of DEGs in Group C and Group N. (**B**–**E**) GSEA of DEGs in Group C and Group N. (**F**) The pathway of CYTOKINE-CYTOKINE RECEPTOR INTERACTION contains a list of genes.

The gene expression list was used as an input file for enrichment analysis. The enrichment of BP was a regulation of lymphocyte activation and T cell activation (Figure 2D). The enrichment of CC was on the external side of the plasma membrane and the secretory granule membrane (Figure 2E). The MFs of DEGs included cytokine activity and cytokine receptor binding (Figure 2F). KEGG enrichment analysis showed cytokine−cytokine receptor interactions and Th17-cell differentiation (Figure 3A). The above enrichment analysis was qualitative analysis, and the input variable of GSEA was gene expression, which could be used for quantitative analysis of the enrichment pathway. The enrichment pathways with higher scores were JAK_STAT_SIGNALING_PATHWAY, T_CELL_RECEPTOR_SIGNALING_PATHWAY, CD8_TCELL_VS_BCELL_NAIVE_UP and CYTOKINE_CYTOKINE_RECEPTOR_INTERACTION (Figure 3B–F) (Table 2).

**Figure 4:**
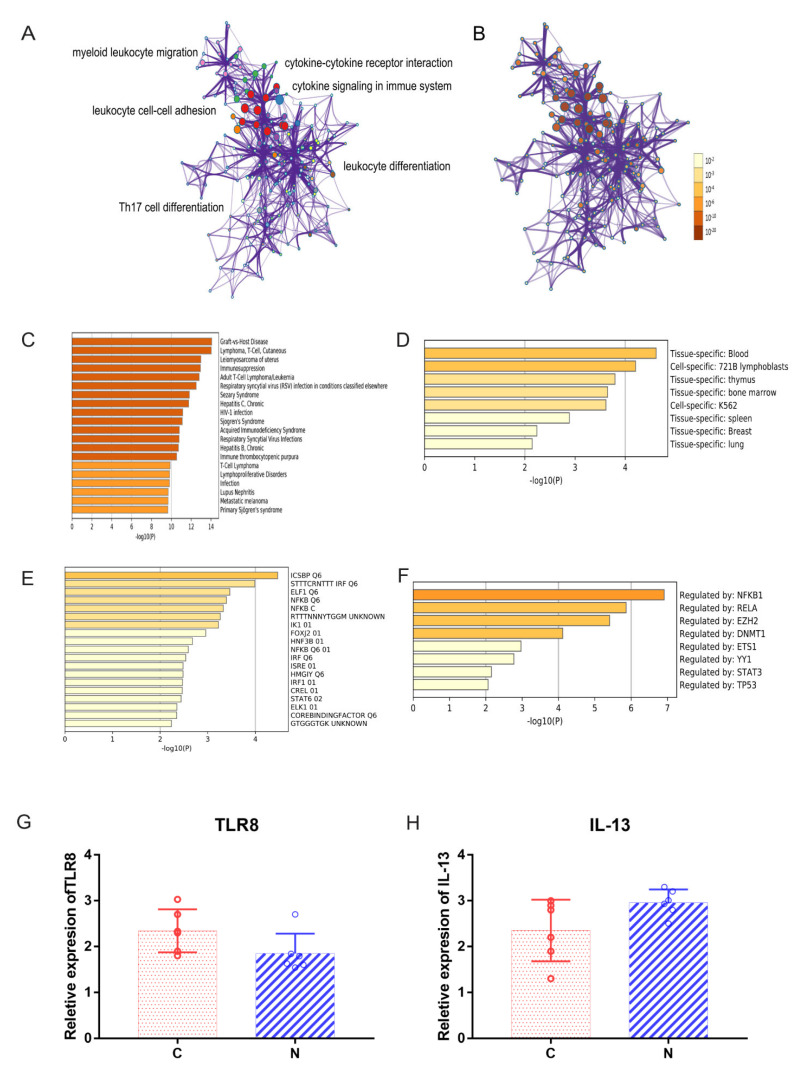
(**A**) Metascape analysis. We selected a subset of representative terms from this cluster and converted them into a network layout. More specifically, each term is represented by a circle node, where its size is proportional to the number of input genes falling into that term, and its color represents its cluster identity (i.e., nodes of the same color belong to the same cluster). Terms with a similarity score > 0.3 are linked by an edge (the thickness of the edge represents the similarity score). The network was visualized with Cytoscape (v3.1.2) with a “force-directed” layout and edge bundled for clarity. One term from each cluster was selected to have its term description shown as a label. (**B**) The same enrichment network has its nodes colored by *p* value, as shown in the legend. The darker the color, the more statistically significant the node is (see legend for *p* value ranges). (**C**) GO terms were statistically enriched by Metascape analysis. (**D**) Tissue- and cell-specific genes were statistically enriched by Metascape analysis. (**E**,**F**) Transcription factors were statistically enriched by Metascape analysis. (**G**,**H**) Relative expression levels of TLR8 and IL-13 by RT–qPCR analysis.

**Figure 5:**
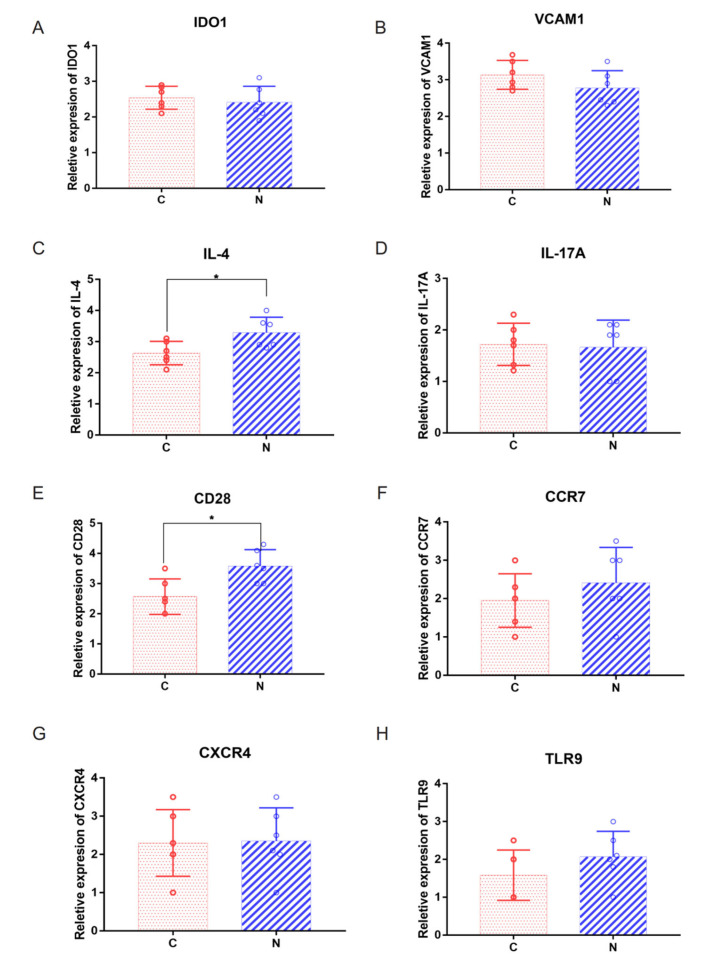
(**A**–**H**) Relative expression levels of IDO1, VCAM1, IL-4, IL-17A, CD28, CCR7, CXCR4 and TLR9 by RT–qPCR analysis. * *p* < 0.05, Group N compared C.

We first identified all statistically enriched terms, and accumulative hypergeometric *p* values and enrichment factors were calculated and used for filtering. The remaining significant terms were then hierarchically clustered into a tree based on Kappa-statistical similarities among their gene memberships. Then, a kappa score of 0.3 was applied as the threshold to cast the tree into term clusters. Metascape analysis also showed that the functions of DEG enrichment were cytokine–cytokine receptor interactions and cytokine signaling in the immune system (Figure 4A). The same enrichment network had its nodes colored by the *p* value (Figure 4B). The differentially expressed gene enrichment in diseases, transcription factors and GO enrichment analysis is shown in Figure 4C–F. GO terms were statistically enriched by Metascape analysis, including graft vs. host disease and lymphoma, T cell and cutaneous (Figure 4C). Tissue-specific and cell-specific genes were statistically enriched by Metascape analysis, including blood and 721B lymphoblasts (Figure 4D). Transcription factors were statistically enriched by Metascape analysis, including NFKB1, RELA and EZH2 (Figure 4E,F). Hub genes were identified by RT–qPCR (Figure 4G,H and Figure 5A–H), including TLR8, IL-13, IDO1, VCAM1, IL-4, IL-17A, CD28, CCR7, CXCR4 and TLR9. The expression of IL-4 was higher in Group N than in Group C (Figure 5C, *p* = 0.0266). The expression of CD28 was higher in Group N than in Group C (Figure 5E, *p* = 0.0110).

### 2.4. CIBERSORT Immune Cell Analysis and CTD Analysis of Hub Genes CD28 and CD8

**Figure 6:**
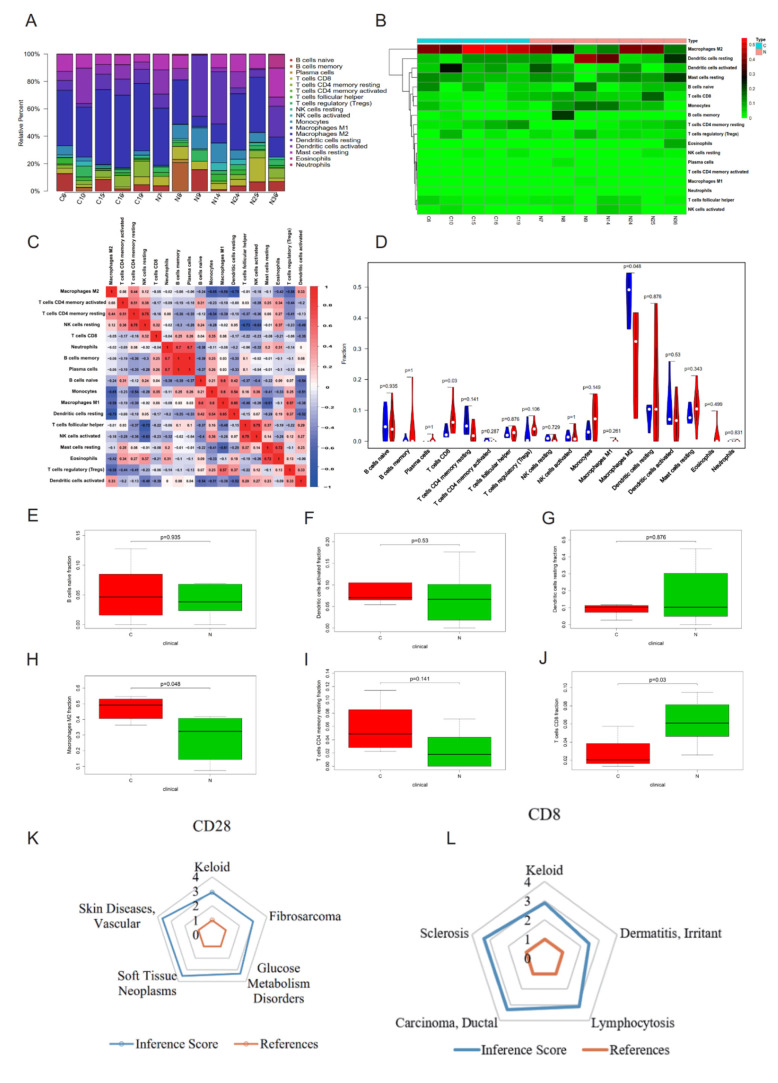
(**A**) Fractions of immune cells between Groups N and C. (**B**) Heatmap of immune cells between Groups N and C. (**C**) A correlational heatmap of immune cells of keloids. (**D**) A violin plot of immune cells between Groups N and C; blue denotes Group C, and red denotes Group N. (**E**–**J**) Quantitative analysis of immune cells by immune infiltration analysis. (**K**,**L**) CD28 and CD8 were further analyzed with the Comparative Toxicogenomics Database.

**Figure 7:**
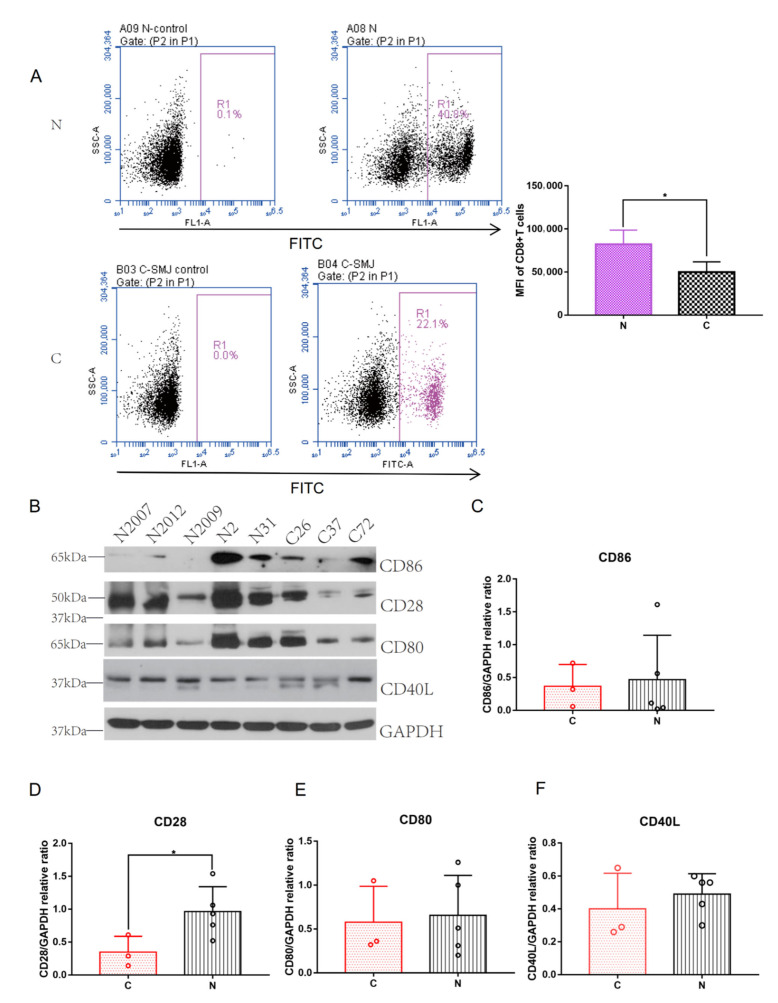
(**A**) CD8^+^ T cells were assayed by flow cytometric analysis between Groups N and C. MFI, Mean Fluorescence Intensity. * *p* < 0.05. (**B**) Western blotting expression of CD86, CD28, CD80 and CD40L between Groups N and C. (**C**–**F**) Quantitative comparison of CD86, CD28, CD80 and CD40L between Groups N and C. * *p* < 0.05.

Costimulatory molecules are important in the activation and effector differentiation of CD8^+^ T cells [26]. CD28 on naive T cells is the most important costimulatory receptor [26,27]. The normalized expression data were converted into cell proportion data using the CIBERSORT algorithm to explore the important immune cells in the pathogenesis of keloids. The bar chart shows that the immune microenvironments of Group N and Group C were not the same, and the cell proportions were unevenly distributed (Figure 6A–C). The violin diagram and box plot showed that there were significant differences in M2 macrophages, which were higher in Group C (Figure 6D,H; *p* = 0.048), and CD8^+^ T cells were higher in Group N (Figure 6D,J; *p* = 0.03). Although the proportions of B cells, dendritic cells and CD4^+^ T cells in the skin immune microenvironment of patients with keloids were higher, there were no significant differences between Group N and Group C (Figure 6D–G,I). CD28 and CD8 were further analyzed with CTD. The CTD database showed that hub genes (CD28 and CD8) were associated with keloids (Figure 6K,L). Flow cytometry showed that the number of CD8^+^ T cells was significantly higher in Group N than in Group C (Figure 7A, *p* = 0.0256).

### 2.5. Abnormal Expression of Costimulatory Signaling Molecules

To identify whether the occurrence of keloids is related to the abnormal activation of CD8^+^ T cells, the expression of important molecules in the CD8^+^ T cell activation pathway was further identified in Groups N and C. The expression of CD28 proteins was higher in Group N than in Group C (Figure 7B,D, *p* = 0.0482). Although the expression levels of CD86, CD80 and CD40L proteins in Group N were not significantly higher than those in Group C, they also showed an upward trend (Figure 7B,C,E,F). CD80, CD86 and CD40L were also elevated in the rabbit ear model (Figure S2). The immunohistochemistry results showed that the expression levels of CD28 (*p* = 0.0106), CD80 (*p* = 0.0226) and CD40L (*p* = 0.0114) were increased in Group N, and there was a significant difference between Group N and Group C (Figure S3). Although CD86 was not significantly higher in Group N, there was an upward trend in Group N. These results may suggest that the abnormal expression of costimulatory molecules (including CD28, CD86, CD80 and CD40L) may lead to the abnormal activation of CD8^+^ T cells, which may be an important pathogenesis of keloids.

### 2.6. CD28 Expressed on the Surface of CD8^+^ T Cells

CD28 is expressed on 90% of CD4^+^ T cells and 50% of CD8^+^ T cells [28]. To determine whether the increase in CD8^+^ T cells was mediated by CD28 on the surface of CD8^+^ T cells rather than other T cells, immunofluorescence was performed by the in situ staining of CD28 and CD8^+^ T cells, and the results showed that the fluorescence signals of CD28 and CD8^+^ T cells overlapped (Figure S4A,B). CD28 was colocalized on CD8^+^ T cells. CD8^+^ T has an upward trend in Group N, but there was no significant difference (Figure S4C). CD28 was significantly higher in Group N than in Group C (Figure S4D, *p* = 0.0003). The fluorescence intensity of CD28 and CD8^+^ T cells in Group N was significantly higher than that in Group C (Figure S4E, *p* = 0.0051). There was an increase of CD8^+^CD28^−^ lymphocytes in keloid tissues compared to normal skin suggesting the occurrence of the CD8^+^ Tregs phenotype (Figure S4F). The expression of CD28 and CD4 had no significant difference between the two groups (Figure S4G). This suggests that the increased expression of CD28 in the skin tissues of patients with keloids may be associated with the abnormal increase in CD8^+^ T cells.

### 2.7. CD8^+^ T Cells and Keloid-Associated Fibroblast Coculture

**Figure 8:**
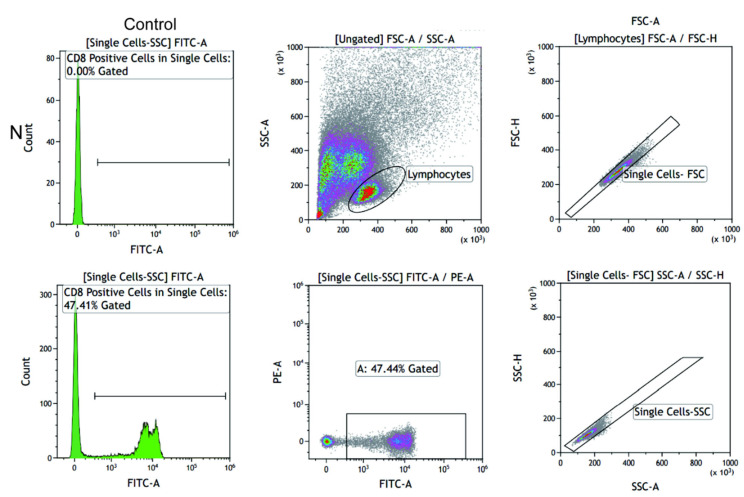
CoraLite^®^488 Anti-Human CD8 (SK1) antibody was used to perform fluorescence-activated cell sorting (Automatic Flow Cytometer MA900, Sony, Tokyo, Japan) in Group N.

**Figure 9:**
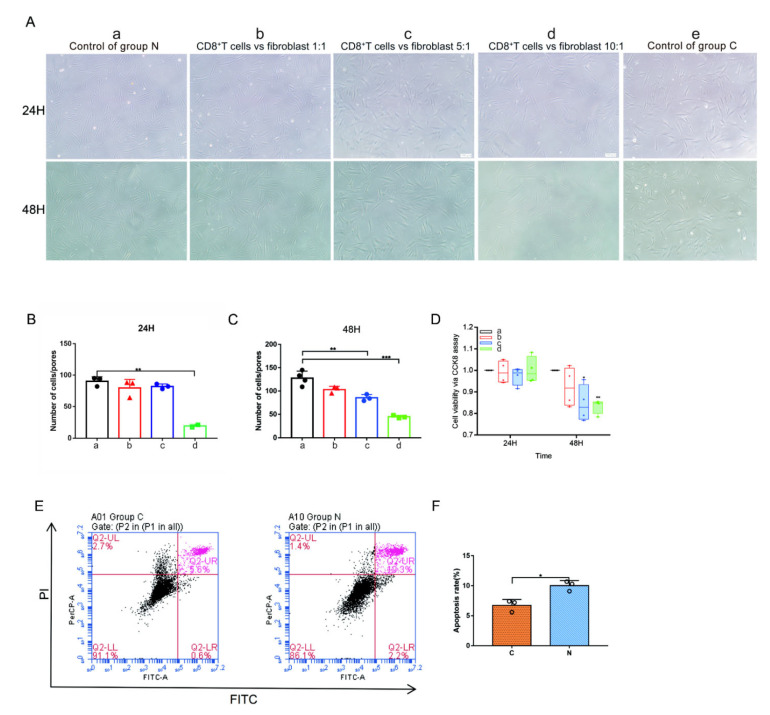
Fibroblasts from patients with keloids were cocultured with CD8^+^ T cells in a Transwell suspension noncontact coculture system. The number of fibroblasts analyzed was measured by microscopy at 100×, n = 3. (**A**) The sorted CD8^+^ T cells and fibroblasts isolated from keloid tissue were cocultured in a Transwell suspension noncontact coculture system. In Group a, only fibroblasts from keloid patients were cultured. In Group b, the ratio of CD8^+^ T cells to fibroblasts was 1:1. In Group c, the ratio of CD8^+^ T cells to fibroblasts was 5:1. In Group d, the ratio of CD8^+^ T cells to fibroblasts was 10:1. The number of fibroblasts growing in Group d was significantly reduced compared with that in Group a at 24 h and 48 h. In Group e, only fibroblasts from cosmetic patients were cultured. (**B**) The numbers of fibroblasts growing in Groups a–d at 24 h. (**C**) The numbers of fibroblasts growing in Groups a–d at 48 h. (**D**) The measure of cell viability via CCK8 assay at 24 h and 48 h. (**E**,**F**) The apoptosis rate (%) was measured by a CoraLite^®^488-Annexin V and PI Apoptosis Kit. Group C: Fibroblasts without coculture at 48 h. Group N: Fibroblasts from patients with keloids cocultured with CD8^+^ T cells directly at 48 h. The ratio of CD8^+^ T cells/fibroblasts in Group C was 5:1. * *p* < 0.05, ** *p* < 0.01, *** *p* < 0.001.

To clarify the effect of CD8^+^ T cells on fibroblasts, a coculture experiment was adopted for further exploration. We extracted PBMCs from patients with keloids by density gradient centrifugation and sorted CD8^+^ T cells by flow cytometry (Figure 8). The sorted CD8^+^ T cells and fibroblasts isolated from keloid tissue were cocultured in a Transwell suspension noncontact coculture system. In Group a, only fibroblasts were cultured. In Group b, the ratio of CD8^+^ T cells to fibroblasts was 1:1. In Group c, the ratio of CD8^+^ T cells to fibroblasts was 5:1. In Group d, the ratio of CD8^+^ T cells to fibroblasts was 10:1. The number of fibroblasts growing in Group d was significantly reduced compared with that in Group a at 24 h (Figure 9A,B, *p* = 0.0010). The numbers of fibroblasts growing in Groups c (Figure 9A,C, *p* = 0.0073) and d (Figure 9A,C, *p* = 0.0003) were significantly reduced compared with that in Group a at 48 h. Cell viability was measured via a CCK8 assay (Figure 9D). The viability of fibroblast growth in Group c was significantly reduced compared with that in Group a at 48 h (Figure 9D, *p* = 0.0110). The viability of fibroblast growth in Group d was significantly reduced compared with that in Group a at 48 h (Figure 9D, *p* < 0.0001).

To explore the mode of CD8^+^ T cells influencing fibroblasts, in addition to the Transwell suspension noncontact coculture system, we also observed the effect of CD8^+^ T cells on fibroblasts through direct contact culture, and CD8^+^ T cells adsorbed around fibroblasts (Figure S5A–E). The numbers of fibroblasts growing in Group b (Figure S5A,C *p* = 0.0006), Group c (Figure S5A,C *p* = 0.0044) and Group d (Figure S5A,C *p* = 0.0005) were significantly reduced compared with that in Group a at 24 h. The viability levels of fibroblast growth in Group c (Figure S5E, *p* = 0.0022) and Group d (Figure S5E, *p* = 0.0003) was significantly reduced compared with that in Group a at 24 h. The number of fibroblasts growing in Group d (Figure S5A,D *p* = 0.0118) was significantly reduced compared with that in Group a at 48 h. The viability levels of fibroblast growth in Group b (Figure S5E, *p* = 0.0001), Group c (Figure S5E, *p* < 0.0001) and Group d (Figure S5E, *p* < 0.0001) were significantly reduced compared with Group a at 48 h. Furthermore, apoptosis of fibroblasts increased after coculture with CD8^+^ T cells (Figure 9E,F, *p* = 0.0114).

### 2.8. Neural Network Model of Keloids

**Figure 10:**
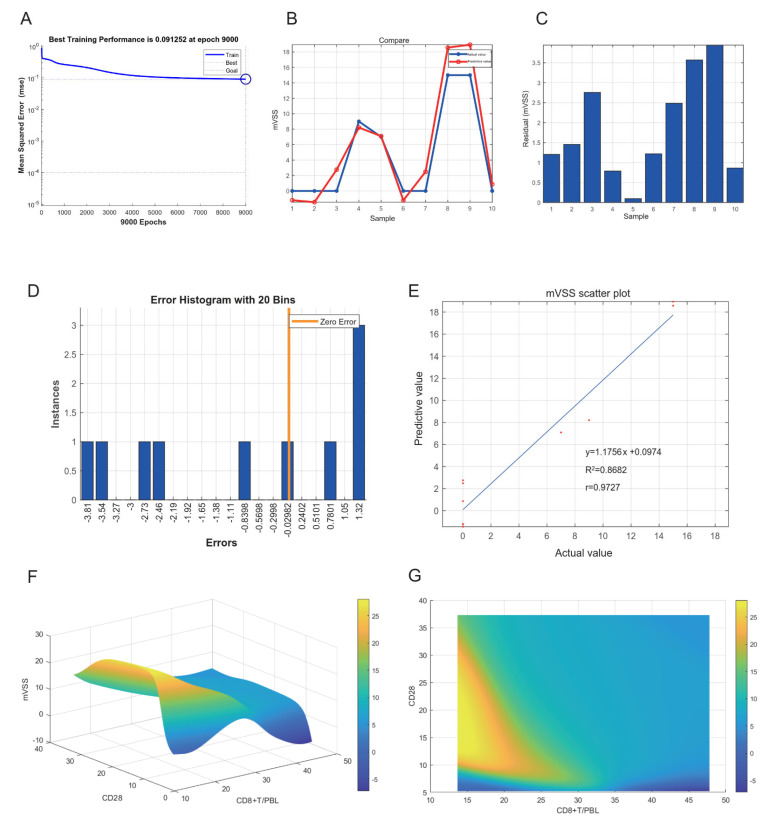
(**A**) The neural network model for predicting the severity of keloids. The best training performance was 0.091252 at epoch 9000. (**B**) The predictive value of the data was verified against the actual value. (**C**) Absolute error diagram between the predicted and actual values of the data. (**D**) Error distribution map between the predicted and actual values of the data. (**E**) Correlation scatter plot of mVSS. y = 1.1756x + 0.0974, R^2^ = 0.8682, r = 0.9727. (**F**,**G**) The high-risk warning range of CD28 and CD8^+^ T/PBL at the planform and three-dimensional levels.

To explore the clinical value of CD28 and CD8^+^ T cells, the expression of CD28 and CD8^+^ T cells were incorporated into the neural network model. The expression of CD28 via the IF score was used as the input layer. CD8^+^ T cells/peripheral blood lymphocytes (PBLs) via flow cytometry were used as the input layer. mVSS was used to determine the severity of the keloid as the output layer. Thirty samples were used as training sets and ten samples were used as verification sets. After the training sets were trained, the neural network model was considered successfully constructed. The best training performance was 0.091252 at epoch 9000 (Figure 10A). Verification sets were used to verify the training effect of the neural network model. The predicted value was basically consistent with the actual value (Figure 10B,C). The error diagram also shows that the error was acceptable (Figure 10D). The predicted value was fitted to the actual value, and the correlation coefficient was 0.9727 (Figure 10E). Based on the successful construction of the neural network model, we can speculate that the expression of CD28 and CD8^+^ T cells may be predictors of the severity of keloids (Figure 10F). The three-dimensional diagram can well show the relationship between the input and output layers. The high-risk early warning indicator for CD28 was 10–34, and the high-risk predictive indicator for CD8^+^ T cells was 13–28 (Figure 10G).

## 3. Discussion

There are tumor-like symptom characteristics in keloids, as they have the ability to expand unchecked, invade surrounding normal tissue and thrive, but they do not have the ability of tumors to metastasize far away [1,2,18]. Patients with keloids often suffer from severe pain and itching. Although there have been many studies on the pathological mechanism of keloids, the pathogenesis mechanism of keloids is still not clear. Keloid development may be the result of the interaction between fibroblasts and immune cells in the surrounding microenvironment [29]. During this process, immune-related genes may be abnormally expressed in primary tissue, which gives rise to an imbalance in the immune microenvironment and ultimately results in hyperplasia of keloids. Determining the major immune cells in this microenvironment and their roles in keloid development will help to illuminate the immune mechanism involved in keloid development. Current research mainly focuses on macrophages and Treg cells [1,2,18]. Tregs inhibit the production of TGF-β by releasing IL-10 [20]. The proportion of Tregs in the keloid dermis was low, which may be the reason for the proliferation of keloids. Tregs can reduce the expression of type I collagen and TGF-β mRNA and inhibit the proliferation of keloid fibroblasts. Macrophages can also increase the expression of Foxp3 to promote the differentiation of Tregs [28]. However, the role of CD8^+^ T cells in keloid development has not been fully elucidated.

The occurrence and development of tumors are the result of the mutual influence and coevolution of tumor cells and surrounding immune cells [30]. Different subtypes of macrophages, CD8^+^ T cells, Tregs and other immune cells infiltrate the tumor microenvironment and regulate it by secreting cytokines. T cells express IL-2 and IFN-γ receptors stimulated by the corresponding cytokines produced by CD4^+^ Th1 cells to activate CD8^+^ cytotoxic T cells and have a killing effect on tumor cells [31]. CD8^+^ T cells activated by tumor immunotherapy mainly induce cell death through perforin granzyme and Fas–Fas ligand pathways to suppress tumor growth [32]. In addition, interferon gamma released by CD8^+^ T cells downregulates the expression of SLC3A2 and SLC7A11, impairing the uptake of cystine by tumor cells and thus promoting lipid peroxidation and iron weakness in tumor cells [33]. Olivo Pimentel V et al. [23] found that CD8^+^ T cells can kill approximately 50% of target tumor cells after 48 h of coculture with tumor cells. Kato T et al. [24] also found that CD8^+^ T cells and cancer-associated fibroblasts were negatively correlated in tumor tissue. The vigorous proliferation ability of keloids has tumor characteristics. The role of CD8^+^ T cells in the tumor microenvironment prompted us to investigate their role in keloid development.

**Figure 11:**
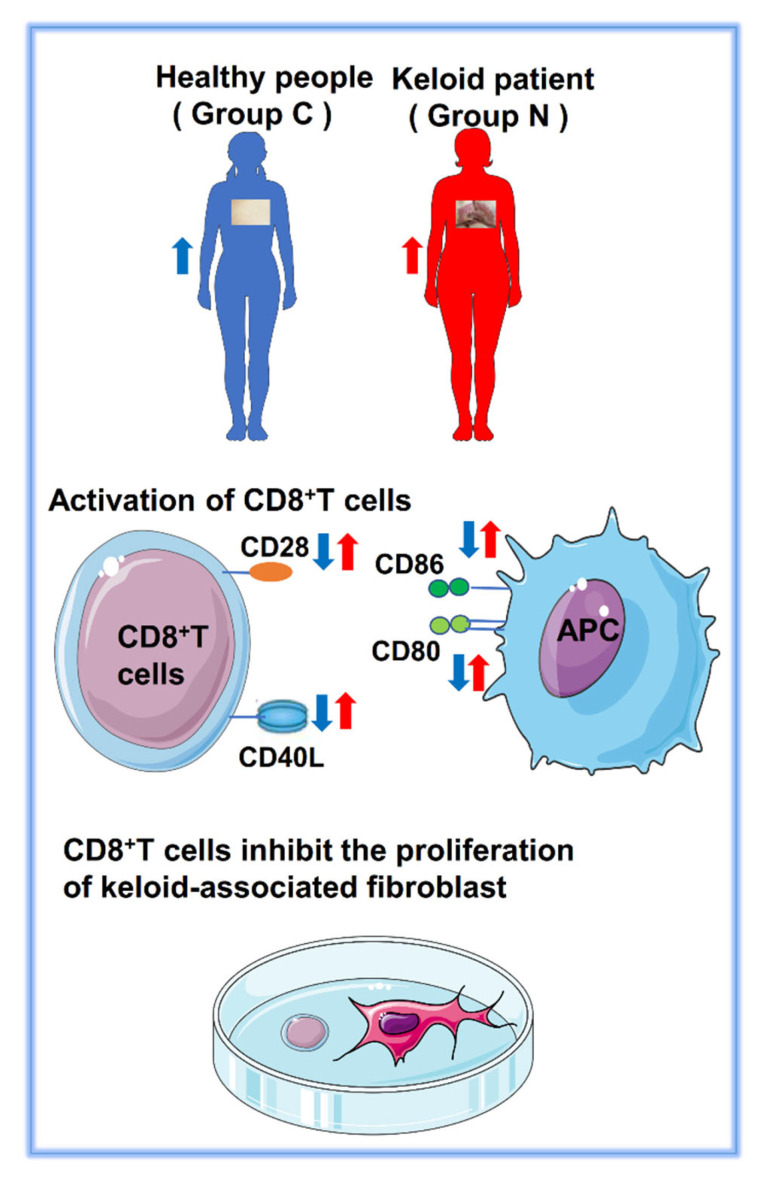
Landscape of this study. The blue arrow represents the expression level of markers in healthy people of Group C. The red arrow represents the expression level of markers in keloid patients of Group N. Arrow up means high expression and arrow down means low expression.

The development of keloids is the result of the mutual influence and coevolution between keloid fibroblasts and surrounding immune cells. CD8^+^ T cells showed the greatest difference in the immune microenvironment between people with keloids and normal people. The abnormal activation of CD8^+^ T cells may be an important pathogenic mechanism of keloids. The activation of naive T cells requires the costimulation of two different extracellular signals: The first signal comes from the interaction and binding of the MHC-antigen peptide complex on the surface of the antigen-presenting cell (APC) with the TCR (including CD4 and CD8) [34,35]. This signal ensures the immune response. The second signal is the interaction and binding between the costimulatory molecules (CD28, CD80, CD86, CD40L and so on) on the surface of APC and the corresponding ligands on the surface of T cells [36]. This signal ensures that the immune response can only occur under the required conditions. Costimulatory molecules are important in the activation and effector differentiation of CD8^+^ T cells [26]. CD28 on naive T cells is the most important costimulatory receptor [26,27]. Anti-CD28 costimulatory T cells have been shown to greatly promote the proliferation and production of IL-2 [37]. Nurieva R et al. demonstrated that the activation of naive T cells in the absence of CD28 resulted in their energy deficiency and effector loss [38]. CD28 must bind to its ligands CD80 (B7-1) and CD86 (B7-2) to induce T-cell activation and differentiation. CD40 expressed by APCs binds to the CD40 ligand expressed by T cells (CD40L) to promote the expression of CD80 and CD86 on APCs. At the same time, CD28 combined with CD80/CD86-upregulated CD40L, resulting in a positive feedback effect [39]. Based on the analysis of the tumor immune-related gene database, the DEGs were compared between patients with keloids (N group) and healthy controls (C group). The expression of costimulatory molecules (CD28, CD80, CD86 and CD40L) in the immune microenvironment of keloids is higher than that in healthy people. Our results may suggest that the abnormal expression of costimulatory molecules (including CD28, CD86, CD80 and CD40L) may exist in the skin tissue of patients with keloids (Figure 11). The expression of IL-4 was significantly increased in Group N compared to C (*p* = 0.0266). During skin wound healing, IL-4 promotes fibroblast chemotaxis and proliferation, myofibroblast differentiation and collagen and extracellular matrix macromolecule production [40]. IL-4 and IL-13 activate the IL-4Rα/STAT6 pathway to promote fibrosis [41]. As an extrinsic factor, IL-4 promotes innate CD8^+^ T cells development in the thymus. It exerts this effect via Eomes upregulation [42]. In the periphery, IL-4 is also a major proliferation stimulator for naïve and memory CD8^+^ T cells in antigen-induced responses [43]. IL-4 stimulation can dramatically promote CCR7 expression by antigen-specific CD8^+^ T cells [44]. The increased expression of IL-4 in the keloid microenvironment may be the factor for CD8^+^ T cells stimulation and proliferation. This then promotes CCR7 expression. An increased CCR7 expression was also observed in this study. The activation of CCR7 increases angiogenesis by upregulating VEGF expression [45]. Obvious angiogenesis with VEGF expression has been reported in the literature [46]. Increased CCR7 expression by CD8^+^ T cells after IL-4 stimulation may be an important factor. Interestingly, the expression of IL-4 was increased in Group N, while the proportion of M2 macrophages was decreased. This may not just be a change caused by M2 macrophages, which may be related to the complex changes in the immune microenvironment, which we will focus on in future research. 

The CIBERSORT algorithm was used to convert gene expression information into immune cell information to explore the key immune cells. The use of the CIBERSORT algorithm to infer the relative proportion of infiltrating immune cells from normalized gene expression data has been reported in explorations of tumor-infiltrating immune cells [47,48]. CD8^+^ T cells in the keloid immune microenvironment may play a regulatory role in the proliferation of keloid-associated fibroblasts. Based on the above research, we found that CD8^+^ T cells have significant differences in the keloid immune microenvironment compared to healthy people. We identified that CD8^+^ T cells drive keloid-associated immunosuppression by flow cytometry and coculture experiments. Although the content of CD8^+^ T cells in the keloid immune microenvironment is high, it may only be the immune reaction state in the face of the disease and has not reached the definite clinical manifestation of inhibiting keloid growth. The reason for this paradox phenomenon needs to be further studied in the future. Methods upregulating such an inhibition may serve as a new direction of targeted therapy, and it is expected to have a therapeutic effect on keloids by increasing the number or function of CD8^+^ T cells in the future. M2 macrophages were significantly increased in Group C compared to N (*p* = 0.048). M2 macrophages are the main macrophage population in the advanced hyperplasia and remodeling stage of keloids [49]. Jin et al. found that the elevation of M1-related genes and proteins in keloid tissues was lower than that of M2-related genes and proteins after skin injury [17]. Our grouping is different from the study, and the M2 macrophage content in normal skin with keloids is lower than that in healthy controls. This may be the reason that the tissue in our study is different from that of the abovementioned studies.

Although our study is based on rigorous bioinformatics analysis and experimental verification, it still has limitations. However, the cells used in the experiment were derived from patients with keloids, and heterogeneity between patients may affect the results. In addition, the inhibitory mechanism of keloid fibroblasts produced by CD8^+^ T also needs further exploration.

## 4. Methods

### 4.1. Patients

This study was approved by the Medical Ethics Committee of Peking Union Medical College Hospital (Medical Ethics Number: JS-2907). All participants provided written informed consent. From June 2019 to February 2021, a total of 53 patients diagnosed with keloid (N group) and 39 patients with cosmetic surgery (C group) were enrolled in this study (Table S2). The mean ± SD age was 34.2 ± 13.0 years. The female-to-male ratio was 14:9. Among them, 12 patients were enrolled for skin tissue high-throughput gene sequencing, including 7 normal skin tissues around keloid tissue (group N) and healthy skin tissue from cosmetic surgery (group C). Skin tissue from cosmetic surgery patients was removed during the operation. Skin tissue from patients with keloids was normal skin tissue 2–3 mm from keloids that was removed during surgery (Table 1). Forty patients were enrolled to construct the neural network model (26 in Group N, 14 in Group C). The other patients were enrolled for subsequent skin tissue experimental verification, HE or immune staining, Western blotting and cell culture (20 patients in each group). Their method of obtaining skin tissue was the same as that for gene sequencing. The modified Vancouver Scar Scale (mVSS) [50] was used to assess the severity of each patient’s keloid(s), according to their manifestation. None of the patients had systemic disease and were taking medication or receiving treatment.

### 4.2. Fibroblasts from Patients with Keloids Cocultured with CD8^+^ T Cells

A coculture experiment of CD8^+^ T cells/fibroblasts from patients with keloids was constructed by a Transwell suspension noncontact coculture system (Millicell^®^ Cell Culture Inserts, 12-well hanging inserts, MCHT12H48, Merck, Darmstadt, Germany). CD8^+^ T cells were activated with beads coated with monoclonal antibodies (mAb) against CD3 and CD28 (Dynabeads CD3/CD28 T-cell Expander; Dynal Biotech–Invitrogen) (bead-to-cell ratio 1:1) and recombinant human interleukin-2 (rhIL-2) (20 units/mL; PeproTech, State of New Jersey, USA). Fibroblasts were cultured in 12-well plates (Thermo Fisher, Waltham Mass, USA), with 1 × 10^4^ cells per well in Group a, by serum starvation overnight. In Group b, the ratio of CD8^+^ T cells to fibroblasts was 1:1. CD8^+^ T cells were cultured in Transwell cells at 1 × 10^4^ per well and fibroblasts were cultured in 12-well plates at 1 × 10^4^ per well. The ratio of CD8^+^ T cells/fibroblasts in Group c was 5:1, and that of CD8^+^ T cells/fibroblasts in Group d was 10:1. Plant hemagglutinin-P 10 μg/mL (P8092, Soleibao Biotechnology Co., Ltd., Beijing, China) was added to RPMI 1640 medium to maintain the in vitro culture of the sorted CD8^+^ T cells. They were incubated in an incubator at 37 °C and 5% CO_2_. To explore the mode of CD8^+^ T cells influencing fibroblasts, in addition to the Transwell suspension noncontact coculture system, we observed the effect of CD8^+^ T cells on fibroblasts through direct contact culture. Other cultivation methods were consistent with the above description.

### 4.3. Neural Network Model

The neural network model is built in MatLab (Version 9.2.0.538062, MathWorks, Natick, MA, USA), and the method used for its construction is the BP algorithm (multilayer feedforward neural network model) for fitting. First, the original data are normalized so that they are distributed between [0, 1]. Then, the normalized data of each group are randomly divided into a training set and verification sets at a ratio of 3:1. The training set is used to build the model, and the prediction set is used to test the model. The number of input neurons in the neural network model is the number of indicators that enter the neural network model. The hidden layer neuron is set to 5, and the output neuron is set to 1. The newff function is used to create the feedforward neural network. The hidden layer uses the S-type activation function tansig. The output layer uses the linear activation function purelin, and the training function uses trading. The number of network training iterations is 9000 times and set every 1000 times to display the primary error. The target error is 10^−5^ and the learning rate is 0.05, based on experience. The momentum factor mc is 0.9. The model is initialized randomly. The output values of output neurons to patients and healthy controls are set to 1–15, respectively. Patients ≥ 1 are regarded as patients. The expression levels of CD8^+^ T cells/PBL measured by flow cytometry and CD28 measured by immunofluorescence were used as the input values, and mVSS was used as the output value. After the model was established, test samples were used to verify the model. Thirty sample data served as the training set, and 10 sample data served as the verification set for test correction. The training model was used to make the model more stable. The mean ± SD age was 32.3 ± 11.7 years. The female-to-male ratio was 4:3.

### 4.4. Exploration of Rabbit Ear Model Construction

Six two-month-old New Zealand white rabbits were used to establish a rabbit ear model. The Animal Care and Use Committee of the Institute of Laboratory Animals, Chinese Academy of Medical Sciences and Peking Union Medical College (CAMS&PUMC) authorized the experimental ethics agreement. Experimental rabbits (n = 6) were used to construct a rabbit ear model with the left ear and the right ear was the control. Zoletil 50 (50 mg/mL, 3 mg/kg animal body weight, Virbac, French) was used for muscle anesthesia on the hind legs of rabbits. A scalpel was used to cut 1 cubic centimeter of wound. After surgery, 40,000 U penicillin was given for 5 days. Four months later, rabbit left ear scars had bulged and grown out of the original wound area ≥ 0.5 cm, which was considered to be a successful construction of the rabbit ear model (Figure S1).

### 4.5. Statistical Analysis

SPSS 24.0 software was used for statistical analysis. GraphPad Prism 7 software and the R package (Version 3.5.3, R Foundation for Statistical Computing, Vienna, Austria) were used to draw statistical graphs. The unpaired t test was used to compare the differences between two groups. A *p* < 0.05 was considered statistically significant.

Other methods used in this study are summarized in Supplementary File.

## 5. Conclusions

CD8^+^ T cells are the main cells for the tumor-specific immune response and have achieved initial clinical success in several cancers, including melanoma, renal cell carcinoma, and Hodgkin’s lymphoma [51,52]. In this study, we found that CD8^+^ T cells can significantly inhibit fibroblasts, which may be the key to keloid immunotherapy (Figure 9, Figure S5). Both direct and indirect cultures of CD8^+^ T cells and fibroblasts have been found to have significant inhibitory effects on fibroblasts. This is an exciting result and may be used in the treatment of keloids in the future to resolve the problem of the overgrowth and recurrence of keloids. In the early stage of keloid development, the body actively responds to the abnormal growth of keloids, which is manifested by the proliferation of CD28^+^CD8^+^ T cells (Figure S4) and the high expression of immune costimulatory molecules. An increase of CD8^+^CD28^−^ lymphocytes in keloid tissues compared to normal skin suggests the occurrence of the CD8^+^ Tregs phenotype (Figure S4F). This may be the reason why keloids persist for a long time in some patients and cannot be eliminated.

These studies will provide a better research basis for CD8^+^ T cells in the treatment of keloids and may open new and effective treatments. The neural network model, which is a network connected by a large number of processing units [53], is an efficient classification method for data mining. It can simulate the basic characteristics of the brain for training and recognition, which can assist doctors in disease diagnosis, prognosis and risk value prediction. To explore the clinical value of this research, the expression of CD28 and CD8^+^ T cells was incorporated into the construction of a neural network model. Based on the successful construction of the neural network model, we can speculate that the expression of CD28 and CD8^+^ T cells may be a predictor of the severity of keloids. The high-risk early warning indicator for CD28 is 10–34, and the high-risk predictive indicator for CD8^+^ T cells is 13–28. These results may provide a new basis for the precise medical treatment of keloids and the evaluation of keloid prognosis.

## Figures and Tables

**Table 1:** Characteristics of patients for sequencing.

Patients	Age of Onset (Years)	Gender	mVSS ^a^
C6	27	male	0
C10	54	female	0
C15	31	female	0
C16	25	male	0
C19	32	female	0
N7	24	female	11
N8	32	male	10
N9	37	female	10
N14	21	male	9
N24	26	female	11
N25	24	female	9
N36	38	male	12

^a^ mVSS: The Modified Vancouver Scar Scale is used for the descriptive assessment of keloids, including melanin (M), height (H), vascularity (V) and pliability (P).

**Table 2:** Pathway enrichment analysis of DEGs in keloid using GSEA.

Gene Set Name	SIZE	ES	NES	Rank at Max
**Upregulated**				
KEGG_EPITHELIAL_CELL_SIGNALING_IN_HELICOBACTER_PYLORI_INFECTION	61	0.563	1.525	3156
KEGG_BASE_EXCISION_REPAIR	31	0.459	1.522	6082
KEGG_ANTIGEN_PROCESSING_AND_PRESENTATION	57	0.653	1.519	2554
KEGG_T_CELL_RECEPTOR_SIGNALING_PATHWAY	101	0.605	1.510	3648
KEGG_TOLL_LIKE_RECEPTOR_SIGNALING_PATHWAY	88	0.702	1.508	2235
KEGG_PATHOGENIC_ESCHERICHIA_COLI_INFECTION	45	0.499	1.505	4454
**Downregulated**				
KEGG_DILATED_CARDIOMYOPATHY	86	−0.524	−1.434	1865
KEGG_HYPERTROPHIC_CARDIOMYOPATHY_HCM	80	−0.499	−1.407	2006
KEGG_LONG_TERM_POTENTIATION	65	−0.390	−1.300	1772
KEGG_ADHERENS_JUNCTION	72	−0.425	−1.272	2780

ES: Enrichment Score; NES: Normalized Enrichment Score.

## Data Availability

The datasets used and/or analyzed during the current study are available from the corresponding author on reasonable request.

## Supplementary Materials

The following supporting information can be downloaded at: https://www.mdpi.com/article/10.3390/ijms23168862/s1. References [54,55,56,57,58] are cited in the Supplementary Materials.
