# Supplementary material for: The Role of CD28 and CD8+ T Cells in Keloid Development
Source: Int J Mol Sci. 2022 Aug 9;23(16):8862. doi: 10.3390/ijms23168862 (PMC9408754; doi:10.3390/ijms23168862)
Supplement: Supplementary file 1 [file ijms-23-08862-s001.zip › ijms-1742812-supplementary.pdf]

Methods

Exploration of differentially expressed genes (DEGs)

An Oncomine immune response research kit (OIRRA, Thermo Fisher, USA, cat. no. A32881) was used to obtain the gene expression profile and was used according to the manufacturer's protocol. To quantify the samples, a TaqMan® Quantitation kit (Thermo Fisher Scientific, Inc.; cat. No. 4468802) was used. The integrity of the sample was verified by agarose gel electrophoresis. The sample was diluted to 100 pM. An Ion 520™ and Ion 530™ kit (Thermo Fisher Scientific, Inc.; cat. no. A27751) was used for sequencing. The nucleotide length was 100-200 bp and the sequencing direction was nonspecific. The data were analyzed by the R package (version 3.6.1). The screening conditions were p value <0.05, log 2-fold change (FC)> 1.5 or <-1.5. DEGs were imported into Cytoscape [54] to construct protein–protein interactions (PPIs) to clarify the interactions between molecules. Molecular complex detection (MCODE) [55] was used to mine the functional modules in the PPI network. GSEA [56] and Metascape [57] analysis were used to mine the information enriched by GO and KEGG to further predict the molecular mechanism of keloids. GO analysis included biological process (BP), cell composition (CC) and molecular function (MF).

Metascape [57]

Pathway and process enrichment analyses were carried out with the following ontology sources: KEGG Pathway, GO Biological Processes, Reactome Gene Sets, Canonical Pathways, CORUM, TRRUST, DisGeNET, PaGenBase, Transcription Factor Targets, WikiPathways, PANTHER Pathway and COVID-19. All genes in the genome were used as the enrichment background. Terms with a p value < 0.01, a minimum count of 3 and an enrichment factor > 1.5 (the enrichment factor is the ratio between the observed counts and the counts expected by chance) were collected and grouped into clusters based on their membership similarities. More specifically, p values were calculated based on the accumulative hypergeometric distribution. The most statistically significant term within a cluster was chosen to represent the cluster.

Gene Set Enrichment Analysis (GSEA) [56]

GSEA is an analysis of genome-wide expression profile microarray data that compares genes to a predefined set of genes. In this database, known genes are grouped and classified according to multiple functional gene sets, such as chromosome location, established gene set, pattern order, tumor-related gene set and GO gene set. The statistical process includes calculating the enrichment fraction, estimating the significance of the enrichment fraction and correcting multiple hypothesis tests.

Comparative Toxicogenomics Database (CTD)

The Comparative Toxicogenomics Database (CTD; http://ctdbase.org/) includes many interspecies chemical gene/protein interactions and gene–disease relationships. The relationships between keloids and hub genes were analyzed using CTD.

Cell type Identification By Estimating Relative Subsets Of RNA Transcripts algorithm (CIBERSORT) [58]

It is an important step to study the immune microenvironment of keloids to determine the proportion of infiltrating immune cells during keloid development. The purpose of deconvolution is to try to infer information about the proportions of immune cells in complex tissues. The CIBERSORT deconvolution algorithm is a machine learning method based on linear support vector regression (SVR) and highly robust to noise that is analyzed by the R package (Version 3.5.3, R Foundation for Statistical Computing, Vienna, Austria). The algorithm is superior to other methods in terms of noise, unknown mixture content and closely-related cell types. Through the CIBERSORT deconvolution algorithm,

the gene expression information was transformed into the proportion information of immune cells, and the significantly different immune cells could be obtained through visual analysis of histograms, heatmaps and violin maps.

Hematoxylin-eosin staining (HE)

Tissue slices were placed in xylene I for 10 minutes, xylene II for 10 minutes, anhydrous ethanol I for 5 minutes, anhydrous ethanol II for 5 minutes, 95% alcohol for 5 minutes, 90% alcohol for 5 minutes, 80% alcohol for 5 minutes and 70% alcohol for 5 minutes. They were then washed with distilled water. Hematoxylin (Thermo Fisher, USA) was used to stain the nuclei and eosin staining (Thermo Fisher, USA) was used to stain the cytoplasm. The slices were dehydrated. The slices were removed from the xylene to dry slightly and then mounted with neutral gum. A microscope was used for image acquisition and image was analyzed using ImageJ (Version 1.8.0).

Immunohistochemical staining

The skin paraffin sections were deparaffinized in water. The samples were incubated in 3% H_2O_2 at room temperature for 10 minutes and were then rinsed with distilled water for 5 minutes. The cells were blocked with 10% goat serum (Thermo Fisher, USA) and incubated at room temperature for 10 minutes. The primary antibody working solution was added dropwise and incubated at 37 ℃ overnight. Biotin-labeled secondary antibody was incubated at 37 ℃ for 30 minutes. An appropriate amount of alkaline phosphatase-labeled streptavidin working solution was added and incubated at 37 ℃ for 30 minutes. After washing with PBS, DAB (Soleibao Biotechnology Co., Ltd. Beijing, China) chromogen was added and allowed to develop for 15 minutes. After the slices were restained, they were dehydrated and sealed. The expression of CD86 was identified by CD86 antibody (dilution rate = 1:300, 13395-1-AP, Proteintech, Chicago, USA). The expression of CD28 was identified by CD28 antibody (dilution rate = 1:2000, ab243228, Abcam, Cambridge, UK). The expression of CD80 was identified by CD80 antibody (dilution rate = 1:500, 66406-1-Ig, Proteintech, Chicago, USA). The expression of CD40L was identified by CD40L antibody (dilution rate = 1:500, 16668-1-AP, Proteintech, Chicago, USA). The expression of CD4 was identified by CD4 antibody (dilution rate = 1:500, 67786-1-Ig, Proteintech, Chicago, USA). The intensity of positive markers was calculated using ImageJ (Version 1.8.0) to obtain the quantitative results.

Immunofluorescence

The skin tissue block was cut into 0.5 cm^2 pieces, and the tissue was sectioned and fixed. The samples were washed with PBS three times for 5 min each time. Sections were added to the permeabilization agent for 15 min, washed with PBS, and sealed for 30 min. The sections were incubated with primary antibody at room temperature for 1 h and then rinsed with PBS. The sections were incubated with secondary antibody at room temperature for 1 h and were then sealed by dropping sealing tablets (Thermo Fisher, USA). The expression of CD86 was identified by CD86 antibody (dilution rate = 1:500, 13395-1-AP, Proteintech, Chicago, USA). The expression of CD28 was identified by CD28 antibody (dilution rate = 1:1000, ab243228, Abcam, Cambridge, UK). The expression of CD80 was identified by CD80 antibody (dilution rate = 1:500, 66406-1-Ig, Proteintech, Chicago, USA). The expression of CD40L was identified by CD40L antibody (dilution rate = 1:500, 16668-1-AP, Proteintech, Chicago, USA). The expression of CD8 was identified by CD8 antibody (dilution rate = 1:500, 66868-1-Ig, Proteintech, Chicago, USA). The intensity of positive markers was calculated using ImageJ (Version 1.8.0) to obtain the quantitative results.

Reverse transcription–quantitative PCR (RT–qPCR)

Skin tissues were extracted for PCR analysis. The primers used in this study are shown in Table S3. Tissue samples (200 mg) were mixed with TRIzol reagent (Thermo Fisher, USA) to extract total RNA using a one-step method. A reverse transcription kit was used to obtain cDNA (HiScript III 1st Strand cDNA Synthesis Kit, Beijing, China). Then, a qPCR fluorescence kit (Taq Pro Universal SYBR qPCR Master Mix, Beijing, China) was used to quantitatively analyze the expression of the target genes. The results were analyzed with $2^{-\Delta\Delta CT}$. GAPDH was used as an endogenous control.

Western blotting (WB)

The skin tissue was ground at 4 °C and a BCA kit (BCA Protein Assay Kit, Thermo Fisher, USA) was used for protein quantification. The protein sample was boiled to denature it. The sample was then added to the electrophoresis apparatus. When the sample was in the concentrate glue, the voltage was set to 80 V; when it was in the separating glue, the voltage was set to 120 V. The protein was transferred from the gel to a polyvinylidene fluoride (PVDF) membrane, the primary antibody was added dropwise and the samples were incubated overnight at 4 °C. The secondary antibody was added and the samples were incubated at room temperature for 2 h. GAPDH was used as an internal reference. CD86 antibody (dilution rate = 1:1000, 13395-1-AP, Proteintech, Chicago, USA) was used to detect CD86. CD28 antibody (dilution rate = 1:1000, A17447, ABclonal, Boston, USA) was used to detect CD28. A CD80/B7-1 monoclonal antibody (dilution rate = 1:1000, 66406-1-Ig, Proteintech, Chicago, USA) was used to detect CD80. CD40L antibody (dilution rate = 1:1000, 16668-1-AP, Proteintech, Chicago, USA) was used to detect CD40L. GAPDH antibody (dilution rate = 1:1000, GB12002, Servicebio, Wuhan, China) was used to detect GAPDH. The intensity of positive markers was calculated using ImageJ (Version 1.8.0) to obtain quantitative results.

Peripheral blood mononuclear cells (PBMCs)

Peripheral blood (20 ml) from each patient was collected. Then, 15 ml peripheral blood and 15 ml PBS were added to a 50 ml centrifuge tube and mixed by pipetting. A total of 15 ml of lymphocyte separation solution (Tianjin Hao ocean, China) was added slowly from the bottom of the blood cells, taking care not to blow out air bubbles. The samples were centrifuged at room temperature (700 g, 30 minutes). A pipette was used to discard the upper layer of clear solution. Pasteur tubes were used to carefully aspirate the cell layer and try not to suck the lower layer of liquid. The samples were centrifuged at room temperature (400 × g, 10 minutes). Five milliliters of red blood cell lysis buffer (Sigma–Aldrich, USA) was added to a 50 ml centrifuge tube and lysed at 4 °C for 1 minute. The lysis was stopped with 40 ml cold PBS. The samples were centrifuged at 4 °C (400 × g, 10 minutes). The cells were washed twice with 10 ml of LB (PBS containing BSA: 1000 ml DPBS+4 ml 0.5M EDTA+5 g BSA). The samples were centrifuged at 4°C (200 × g, 10 minutes).

Isolation and culture of fibroblasts

Keloid tissues were placed in a sterile petri dish (Thermo Fisher, USA). Then, the keloid epidermis was completely peeled off with sterile scissors and separated into tissue blocks approximately 1 mm in size with sterile scissors. The separated tissues were placed in a 15 mL centrifuge tube with trypsin (Thermo Fisher, USA) at 37 °C for 40 min and then centrifuged at 1000 rpm for 5 min. The remaining tissue was discarded through a 100 μm sterile cell filter (Soleibao Biotechnology Co., Ltd. Beijing, China) and the undigested tissue was discarded. Finally, the cell suspension was mixed and planted in DMEM with 10% FBS, penicillin and 100 U/ml streptomycin. Fibroblasts were passaged 3 times for the experiment.

Flow cytometry

PBMCs were added to 1 mL blocking buffer and incubated at room temperature for 30 min. The concentration of the cell suspension in each tube was 1 x 10^6 cells. CoraLite®488 Anti-Human CD8 (SK1) (CL488-65146, Proteintech, Chicago, USA) antibody was added and incubated at room temperature for 2 h. Then, 1 mL PBS was added. The samples were centrifuged at 150 × g for 5 min. Diluted secondary antibodies were added to the samples and incubated at room temperature for 1 hour. Then, 1 mL PBS was added. The cells were centrifuged at 150 × g for 5 min. The cells were resuspended in 150 μl PBS buffer and the results were analyzed by flow cytometry. CoraLite®488 Anti-Human CD8 (SK1) (CL488-65146, Proteintech, Chicago, USA) antibody was used to perform fluorescence-activated cell sorting (Automatic Flow Cytometer MA900, Sony, Japan). The entire gating strategy is shown in Figures S6–S8.

The skin samples were cut into small pieces, with a diameter of no more than 4 mm, digested overnight with the Whole Human Skin Dissociation Kit (Miltenyi, Germany) to maximize the retention of cells, and then disrupted into a single cell suspension with gentle MACS Dissociator. Single-cell suspensions were stained with flow-through antibodies. CoraLite®488 Anti-Human CD8 (SK1) (CL488-65146, Proteintech, Chicago, USA) and APC Anti-Human CD28 (APC-65099, Proteintech, Chicago, USA) antibody were used.

Cell Counting Kit-8 (CCK-8)

After processing according to different experimental conditions, the proliferation activity of fibroblasts was detected by CCK-8. CCK8 working solution was prepared by mixing CCK-8 reagent (C0037, Beyotime Biotechnology, Shanghai, China) and RPMI 1640 medium in a volume ratio of 1:10. Then, 110 μL of CCK-8 working solution was added to each well, mixed well and incubated for 1-4 h in a 37 °C incubator. Then, 90 μL of supernatant was collected from each well and added to the ELISA plate. A microplate reader was used to detect the absorbance at 450 nm and 630 nm. OD value = OD450-OD630. CCK-8 working solution was used as a blank control. Cell viability% = (OD experimental group-OD blank group)/(OD control group/OD blank group) × 100%.

Apoptosis assay

The CoraLite®488-Annexin V and PI Apoptosis Detection Kit (PF00005, Proteintech, Chicago, USA) provides a quick and easy method to detect apoptosis. Cell apoptosis was induced in accordance with the experimental protocol. Untreated cell samples were used as a negative control. Appropriate samples for single dye staining and compensation were also included. Digestion was performed with EDTA-free trypsin (T1350, Soleibao Biotechnology Co., Ltd. Beijing, China), and then the samples were centrifuged at 300 × g for 5 min at 4 ℃ to collect the cells. The cells were washed twice with ice-cold PBS and centrifuged at 300 × g for 5 min at 4 °C. Then, 1-5×10^5 cells were collected and resuspended in 100 μL of 1× binding buffer. Then, 4-5 μL of CL488-Annexin V and 5 μL of PI working solution were added to each tube. The samples were incubated for 10-15 minutes at room temperature in the dark. After the incubation period, 400 μL of 1X binding buffer (or 1X PBS) was added to each tube. As soon as possible, the stained cells were analyzed on a flow cytometer (BD Accuri C6 Plus 1.0.23.1). CL488-Annexin V was excited by a 488 nm laser, the detection fluorescence emission spectrum was at 530 nm (FITC channel) and the emission spectrum of PI was at approximately 617 nm.

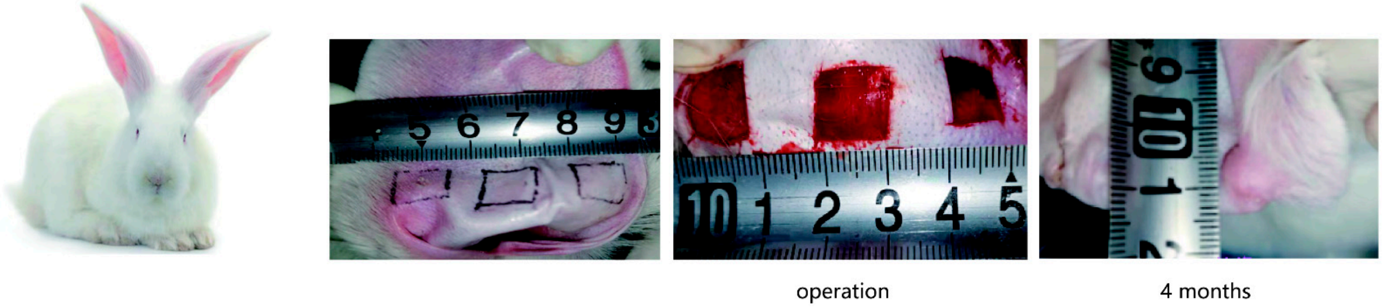

Figure S1. Construction process of the rabbit ear model.

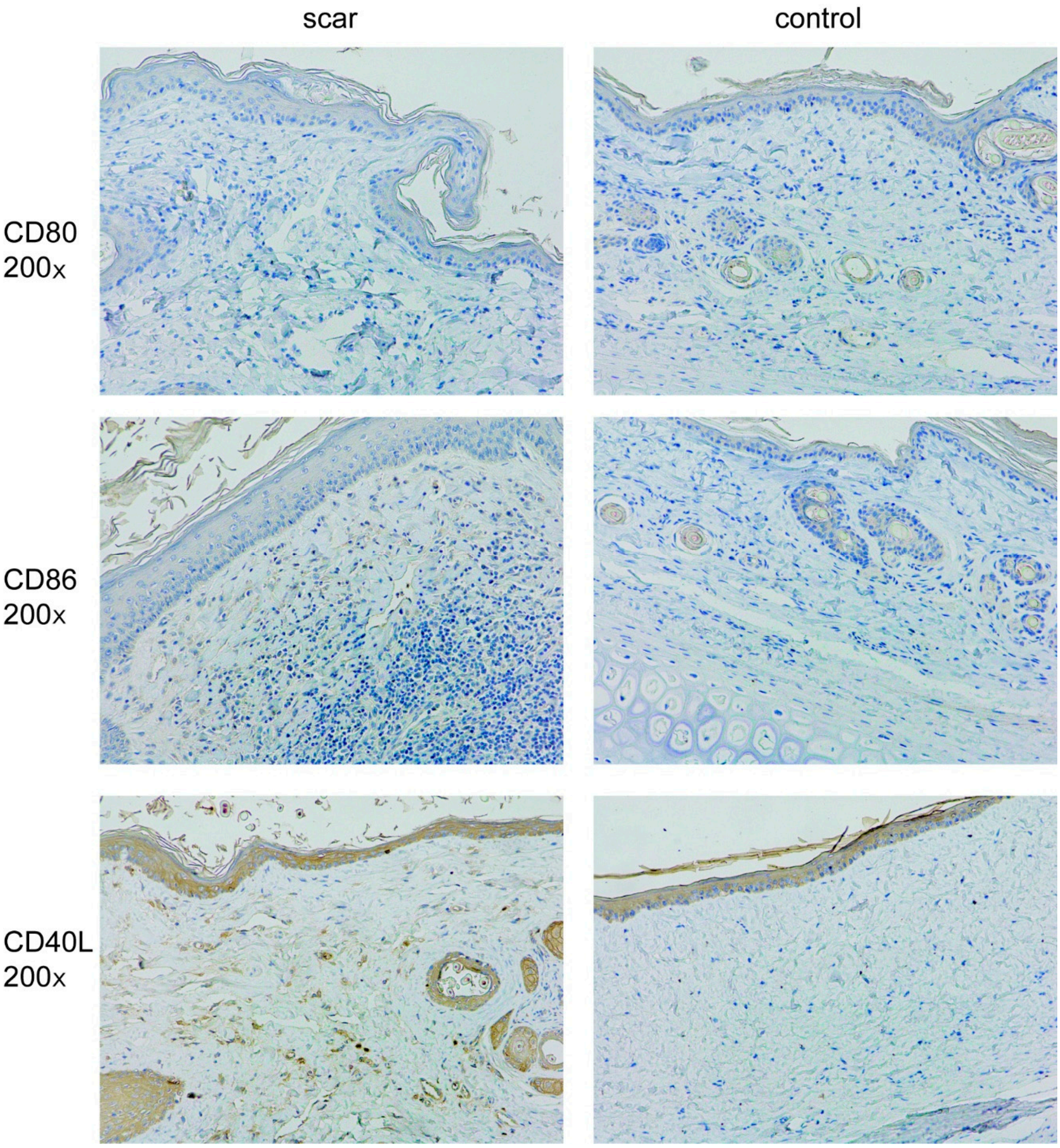

Figure S2. The detection of CD80, CD86 and CD40L in the rabbit ear model by immunohistochemical staining with antibody (Gross appearance, 200x).

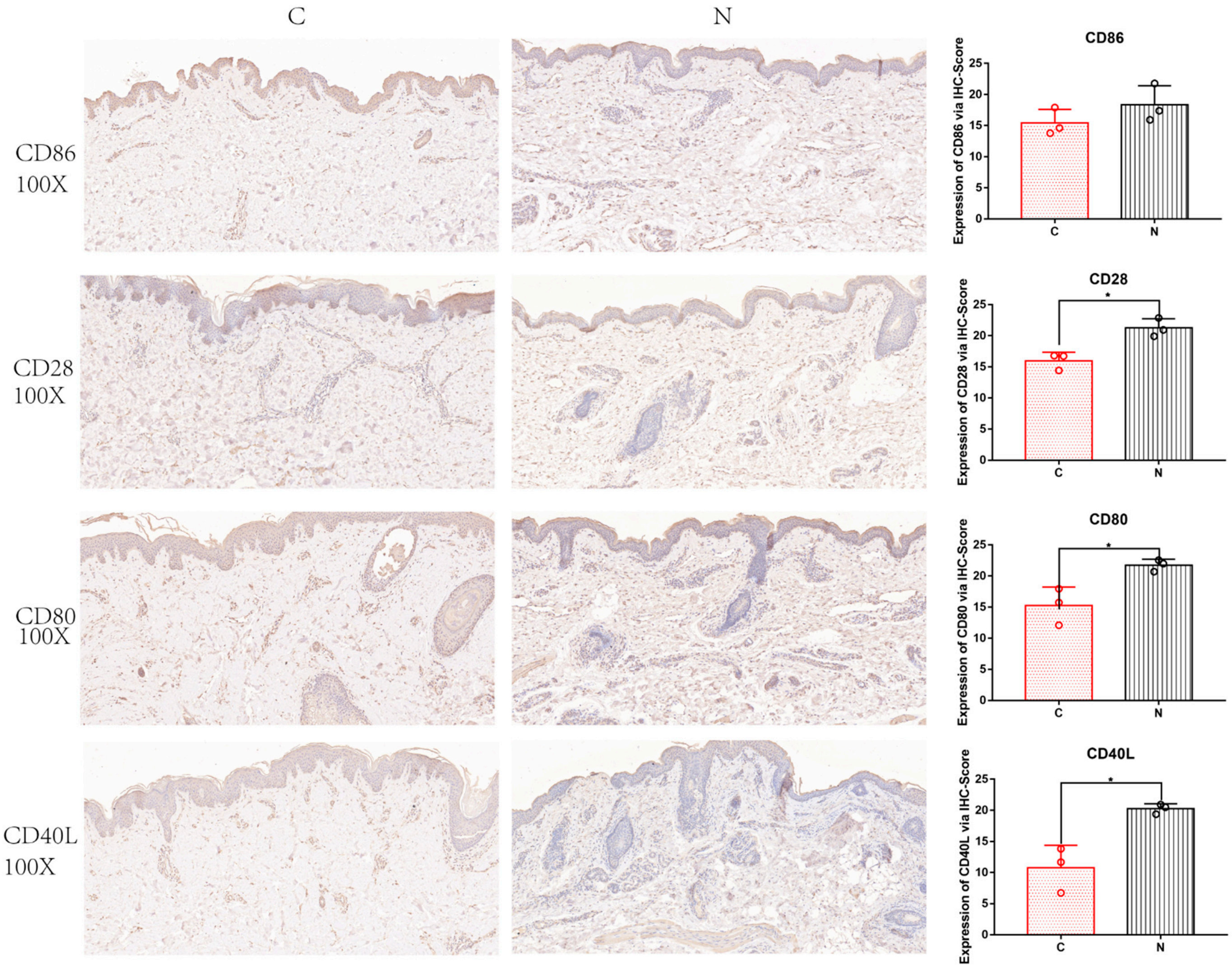

Figure S3. The detection of CD86, CD28, CD80 and CD40L in Groups C and N by immunohistochemical staining with antibodies (Gross appearance, 100x).

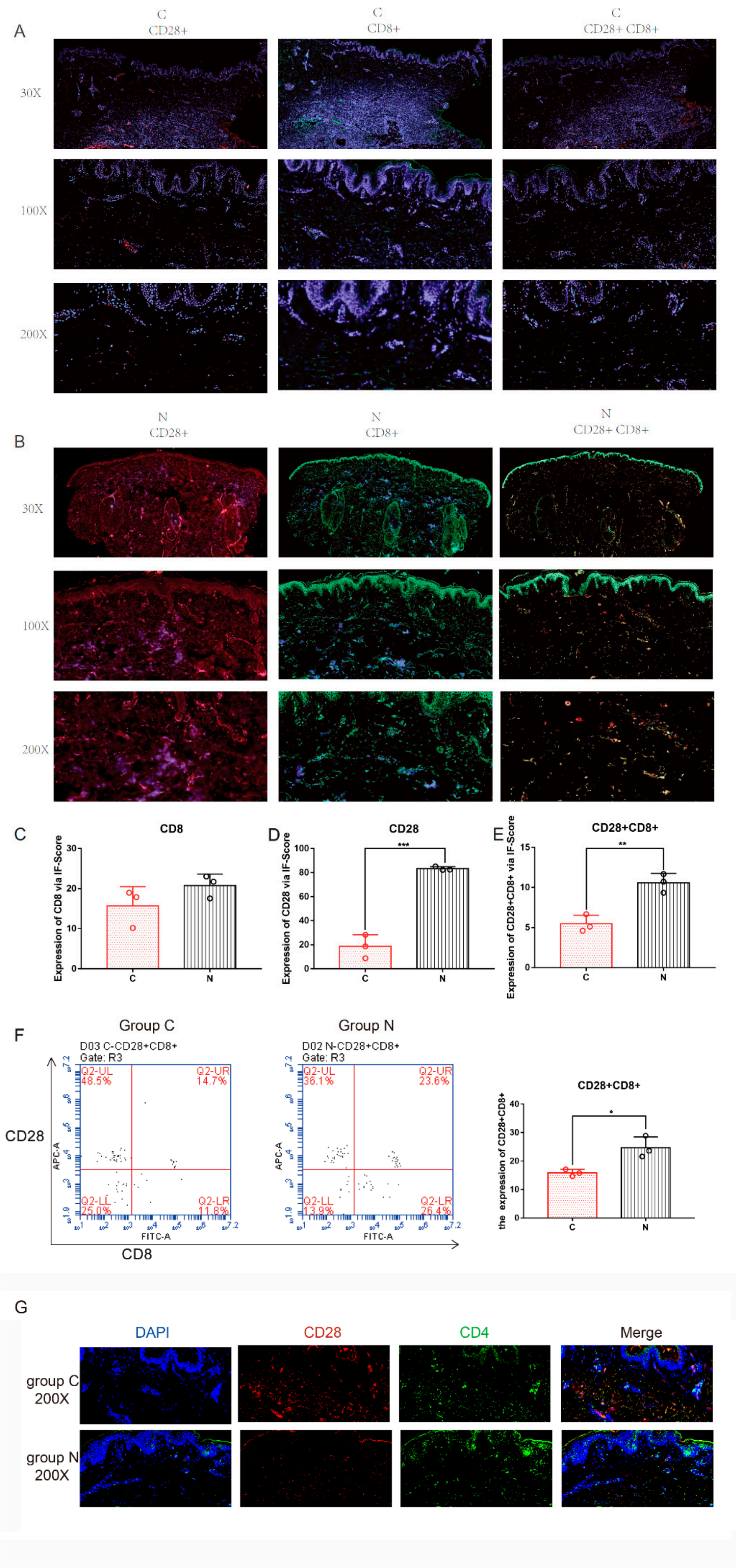
A
C
CD28+
C
CD8+
C
CD28+ CD8+
30X
100X
200X
B
N
CD28+
N
CD8+
N
CD28+ CD8+
30X
100X
200X
C
CD8
Expression of CD8 via IF-Score
C
N
D
CD28
Expression of CD28 via IF-Score
***
E
CD28+CD8+
Expression of CD28+CD8+ via IF-Score
**
F
Group C
Group N
D03 C-CD28+CD8+
Gate: R3
D02 N-CD28+CD8+
Gate: R3
Q2-UL
48.5%
Q2-UR
14.7%
Q2-LL
25.0%
Q2-LR
11.8%
Q2-UL
36.1%
Q2-UR
23.6%
Q2-LL
13.9%
Q2-LR
26.4%
APC-A
FITC-A
CD28
CD8
CD28+CD8+
the expression of CD28+CD8+
*
G
DAPI
CD28
CD4
Merge
group C
200X
group N
200X

Figure S4. (A) The detection of CD28, CD8, CD28 and CD8 content in Group C by immunofluorescence assay. (Gross appearance, 30X, 100X, 200X). (B) The detection of CD28, CD8, CD28 and CD8 contents in Group N by immunofluorescence assay. (Gross appearance, 30X, 100X, 200X). (C-E) Quantitative comparison of CD8, CD28, CD28 and CD8 contents in Groups C and N by immunofluorescence assay. (F) The CD28 expression on CD8⁺ T cells were verified by Flow Cytometry. (G) The detection of CD28 and CD4 in Group C and N by immunofluorescence assay. (Gross appearance, 200X).

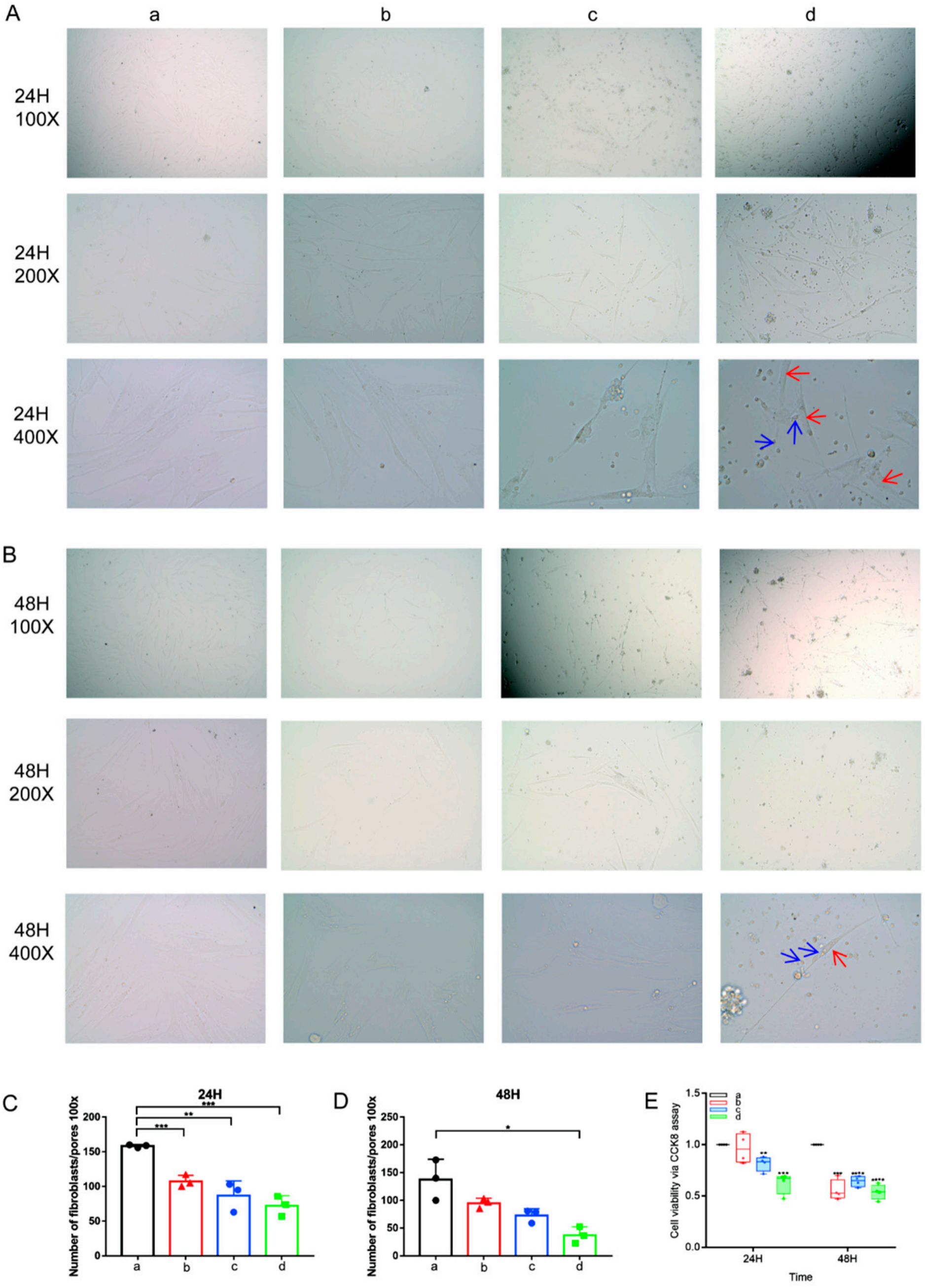

Figure S5. (A-B) Fibroblasts from patients with keloids cocultured with CD8⁺T cells directly at 24 h and 48 h, 100X, 200X and 400X. In Group a, only fibroblasts were cultured. In Group b, the ratio of CD8⁺ T cells and fibroblasts was 1:1. In Group c, the ratio of CD8⁺ T cells to fibroblasts was 5:1. In Group d, the ratio of CD8⁺T cells to fibroblasts was 10:1. (C) The number of fibroblasts was measured by microscopy at 100X, n=3, 24H. (D) The number of fibroblasts was measured by microscopy at 100X, n=3, 48H. (E) The measure of fibroblast viability via CCK8 assay at 24 h and 48 h. The red arrow shows fibroblasts from patients with keloids and the blue arrow shows CD8⁺ T cells.

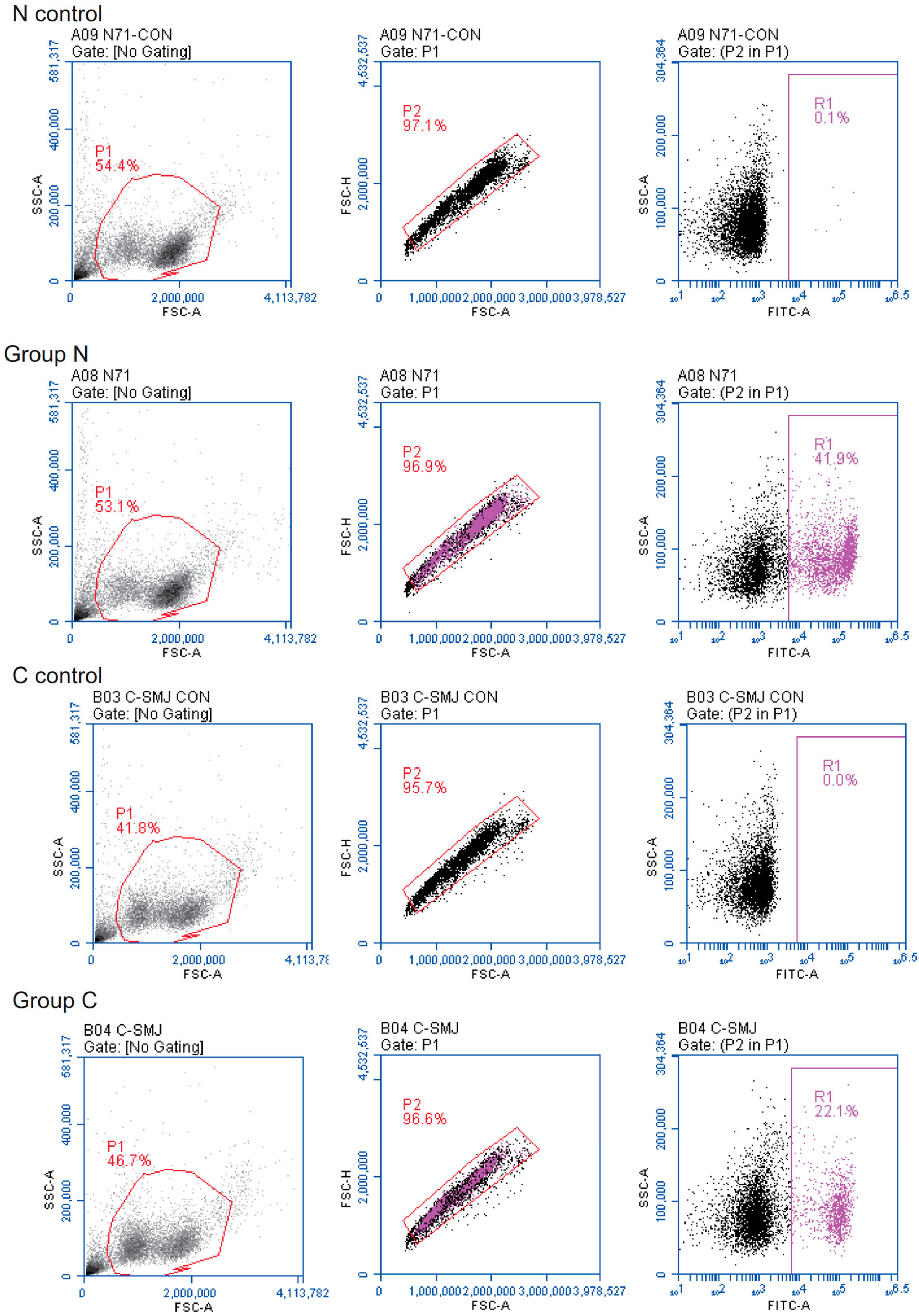
N control
A09 N71-CON
Gate: [No Gating]
P1
54.4%
SSC-A
FSC-A
Gate: P1
P2
97.1%
FSC-H
Gate: (P2 in P1)
R1
0.1%
FITC-A
Group N
A08 N71
Gate: [No Gating]
P1
53.1%
Gate: P1
P2
96.9%
Gate: (P2 in P1)
R1
41.9%
C control
B03 C-SMJ CON
Gate: [No Gating]
P1
41.8%
Gate: P1
P2
95.7%
Gate: (P2 in P1)
R1
0.0%
Group C
B04 C-SMJ
Gate: [No Gating]
P1
46.7%
Gate: P1
P2
96.6%
Gate: (P2 in P1)
R1
22.1%

Figure S6. The entire gating strategy of flow cytometric analysis that CD8⁺ T cells were assayed between Groups N and C.

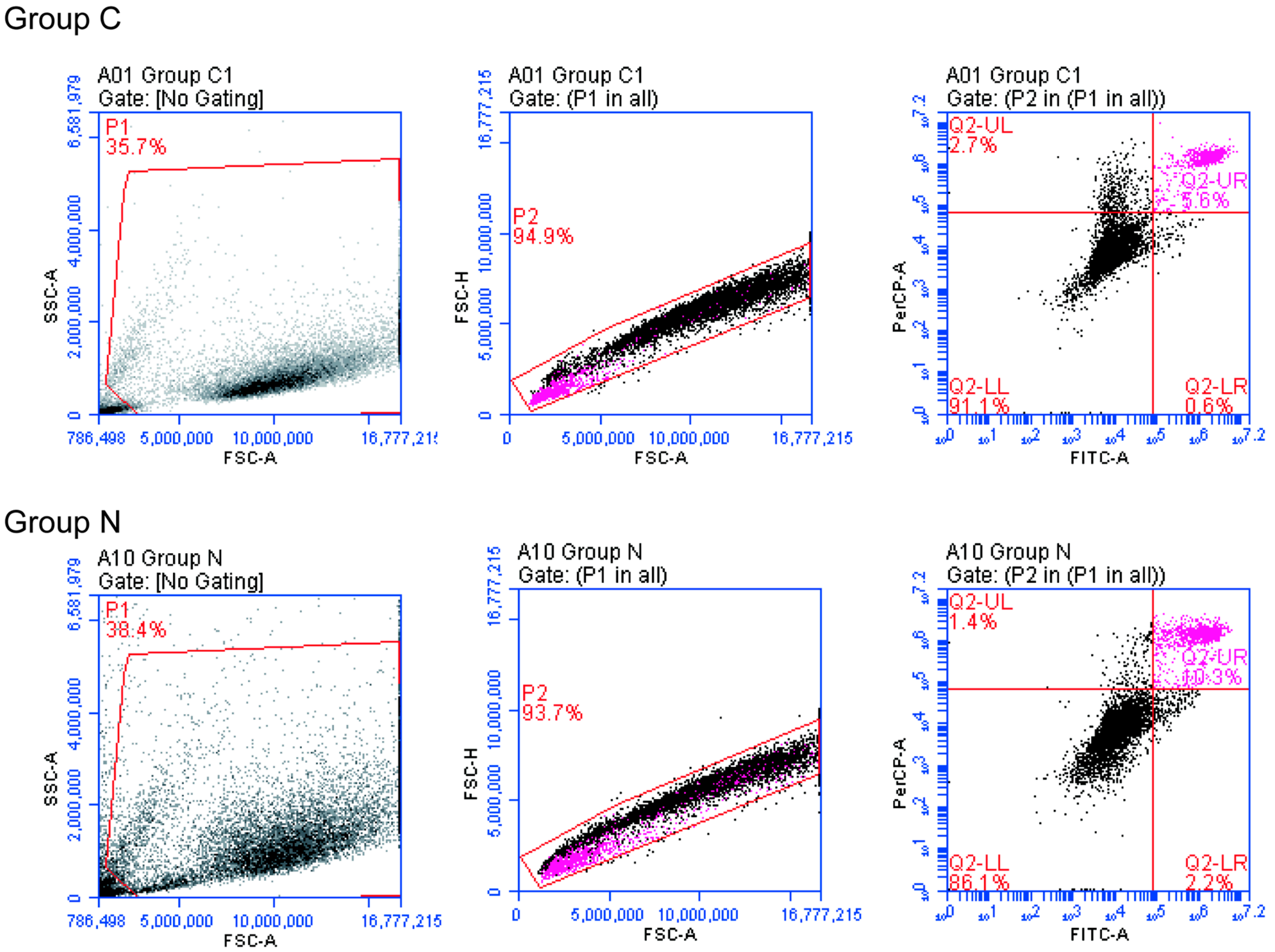

Figure S7. The entire gating strategy of apoptosis detection.

N control

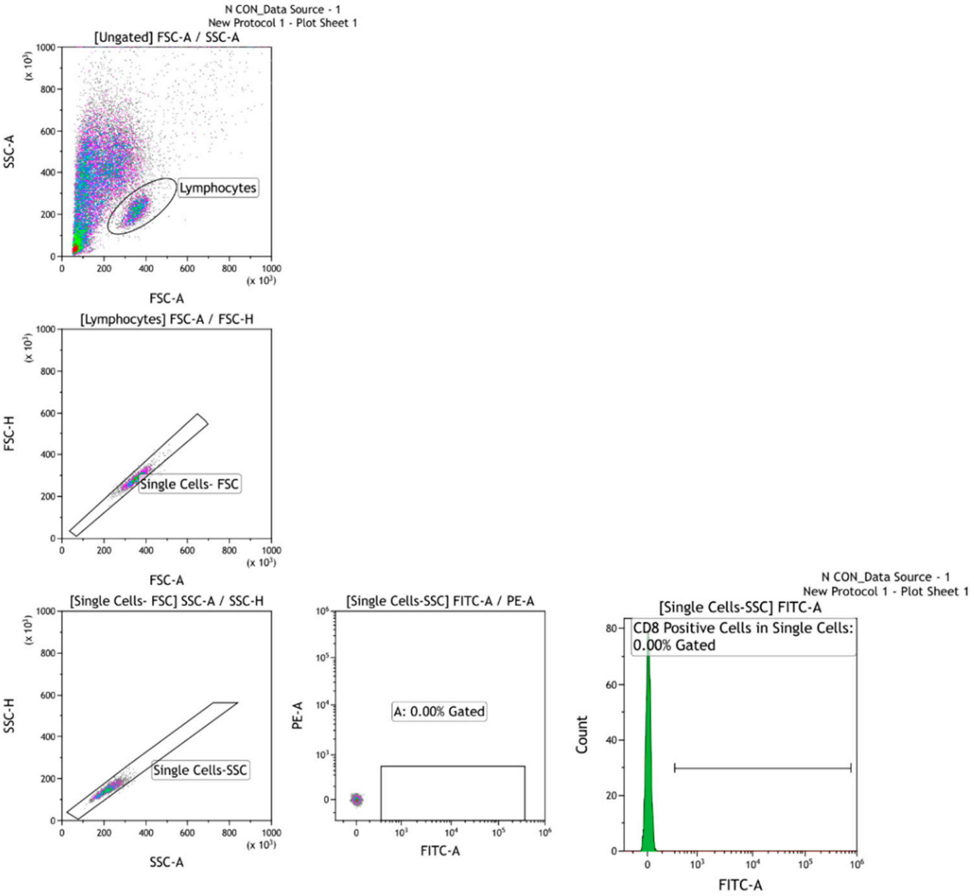

Group N

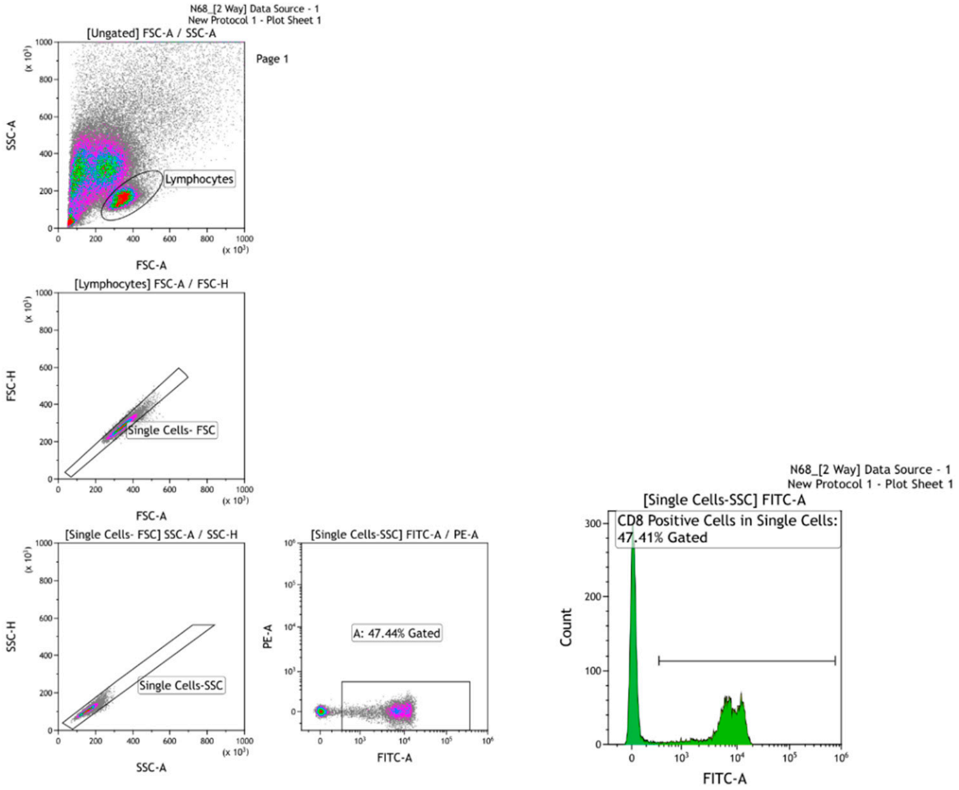

Figure S8. The entire gating strategy of fluorescence-activated cell sorting.

Table S1. Differentially expressed genes between the two groups.

Gene Symbol	Fold Change	P-val	FDR P-val
**Upregulated**			
TIGIT	23.19	0.0105	0.1497
CCL20	20.87	0.0014	0.0685
CCL18	15.39	0.012	0.1497
KLRB1	9.2	0.0109	0.1497
LAMP3	8.61	0.0124	0.1497
PTPRCAP	7.61	0.0485	0.2976
AXL	7.29	0.0066	0.1251
BATF	6.99	0.0196	0.1698
IL7R	6.78	0.0248	0.1951
TYROBP	5.85	0.0218	0.1845
IKZF4	5.24	0.0009	0.0618
CX3CR1	5.17	0.0355	0.2524
CCL22	3.99	0.025	0.1951
CD4	3.79	0.0024	0.0734
TNFAIP8	3.39	0.0022	0.0734
HLA-DPB1	2.77	0.01	0.1497
TWIST1	2.71	0.0365	0.2546
LRP1	2.69	0.0494	0.2977
NFKBIA	2.51	0.0423	0.2719
SRGN	2.44	0.0149	0.1535
CBLB	2.18	0.0302	0.2226
NFATC1	2.13	0.0174	0.1609
AIF1	2.03	0.0289	0.2214
MAPK14	1.93	0.0377	0.2589
DGAT2	1.84	0.0413	0.2719
**Downregulated**			
IDO1	-2.11	0.0424	0.2719
CDKN2A	-3.44	0.019	0.1698
HLA-F-AS1	-3.49	0.0316	0.2288
TNFSF4	-4.27	0.0147	0.1535
TDO2	-4.28	0.0174	0.1609
RB1	-4.69	0.0401	0.2708
KIR2DL2	-4.72	0.0132	0.1497
MAGEA4	-4.72	0.0132	0.1497
CTAG1B	-4.72	0.0132	0.1497
MAGEA12	-4.72	0.0132	0.1497
KIR2DL1	-4.72	0.0132	0.1497
IL21	-4.72	0.0229	0.1887
CEACAM8	-4.72	0.0232	0.1887
CMKLR1	-4.93	0.0138	0.1522
IL4	-5.19	0.015	0.1535
SSX2	-5.26	0.0076	0.1383
GAGE13	-5.26	0.0164	0.1609
MAGEA3	-5.99	0.0048	0.1164
MAGEA1	-6.01	0.0195	0.1698
BUB1	-6.05	0.0058	0.1164
MAGEC2	-6.56	0.0019	0.0685
TNFSF18	-6.84	0.0057	0.1164
TNFSF9	-7.74	0.0059	0.1164

MAGEA10	-8.38	0.0106	0.1497
IFITM2	-10.33	0.0005	0.045
ID2	-10.45	0.0004	0.045
GAGE1	-11.43	0.0171	0.1609
TNFSF14	-16.24	0.0053	0.1164
LCK	-16.35	0.013	0.1497
NTN3	-17.41	0.0006	0.045
BAGE	-25.44	0.0003	0.045
TNFSF13B	-26.19	0.0057	0.1164
GAGE2C	-40.77	0.0084	0.1454
PTEN	-58.97	0.0012	0.0681
NCR1	-71.06	0.0029	0.0814
FUT4	-76.02	3.08×10^{-5}	0.0123
PECAM1	-528.03	0.0302	0.2226
IFNB1	-3905.18	0.0019	0.0685
IFNA17	-9722.53	0.0018	0.0685

Table S2. Clinical features of patients.

Characteristic	Keloid patients	Cosmetic patients	P value
Gender			0.91
male	21 (22.8%)	15 (16.3%)	
female	32 (34.8%)	24 (26.1%)	
Age			0.184
18-35	32 (34.8%)	29 (31.5%)	
36-59	18 (19.6%)	10 (10.9%)	
≥60	3 (3.3%)	0	
Age of onset			/
14-35	42 (45.7%)	/	
36-59	11 (12.0%)	/	
Pathogenic site			/
forebreast	39 (42.4%)	/	
ear	6 (6.5%)	/	
shoulder	8 (8.7%)	/	

Table S3. Primers and their sequences for PCR analysis.

Genes	Sequence (5′–3′)
TLR8-F	ATGTTCCTTCAGTCGTCAATGC
TLR8-R	TTGCTGCACTCTGCAATAACT
IL-13-F	CCTCATGGCGCTTTTGTTGAC
IL-13-R	TCTGGTTCTGGGTGATGTTGA
IDO1-F	GCCAGCTTCGAGAAAGAGTTG
IDO1-R	ATCCCAGAACTAGACGTGCAA
VCAM1-F	GGGAAGATGGTCGTGATCCTT
VCAM1-R	TCTGGGGTGGTCTCGATTTTA
IL-4-F	CCAACTGCTTCCCCCTCTG
IL-4-R	TCTGTTACGGTCAACTCGGTG
IL-17A-F	TCCCACGAAATCCAGGATGC
IL-17A-R	GGATGTTCAGGTTGACCATCAC
CD28-F	CTATTTCCCGGACCTTCTAAGCC
CD28-R	GCGGGGAGTCATGTTCATGTA
CCR7-F	TGAGGTCACGGACGATTACAT
CCR7-R	GTAGGCCCACGAAACAAATGAT

CXCR4-F	ACTACACCGAGGAAATGGGCT
CXCR4-R	CCCACAATGCCAGTTAAGAAGA
TLR9-F	CTGCCTTCCTACCCTGTGAG
TLR9-R	GGATGCGGTTGGAGGACAA

References

54. Shannon, P.; Markiel, A.; Ozier, O.; Baliga, N.S.; Wang, J.T.; Ramage, D. Cytoscape: A Software Environment For Integrated Models Of Biomolecular Interaction Networks. *Genome Res.* **2003**, *13*, 2498–2504.
55. Bader, G.D.; Hogue, C.W. An Automated Method for Finding Molecular Complexes in Large Protein Interaction Networks. *BMC Bioinform.* **2003**, *4*, 2.
56. Canzler, S.; Hackermüller, J. Multigsea: A Gsea-Based Pathway Enrichment Analysis for Multi-Omics Data. *BMC Bioinform.* **2020**, *21*, 561.
57. Zhou, Y.; Zhou, B.; Pache, L.; Chang, M.; Khodabakhshi, A.; Tanaseichuk, O.; Benner, C.; Metascape, S.K.C. Provides A Biologist-Oriented Resource For The Analysis Of Systems-Level Datasets. *Nat. Commun.* **2019**, *10*, 1523.
58. Chen, B.; Khodadoust, M.S.; Liu, C.L.; Newman, A.M.; Alizadeh, A.A. Profiling Tumor Infiltrating Immune Cells With Cibersort. *Methods Mol. Biol.* **2018**, *1711*, 243–259.
